# Autism Spectrum Disorder Induced Pluripotent Stem Cells Display Dysregulated Calcium Signaling During Neural Differentiation

**DOI:** 10.3390/cells14171402

**Published:** 2025-09-08

**Authors:** Abdullah J. AlShawaf, Sarah A. AlNassar, Norah AlGhamdi, Cristiana Mattei, Shiang Y. Lim, Mirella Dottori, Futwan A. Al-Mohanna

**Affiliations:** 1 Department of Physical Therapy, Mohammed Al-Mana College for Medical Sciences, Dammam 34222, Saudi Arabia; a.alshawaf@machs.edu.sa; 2 Department of Physiological Sciences, College of Medicine, AlFaisal University, Riyadh 11533, Saudi Arabia; 3 Department of Cell Biology, King Faisal Specialist Hospital & Research Centre, Riyadh 11211, Saudi Arabia; sarahaln@kfshrc.edu.sa; 4 Biological and Environmental Sciences and Engineering Division, King Abdullah University for Science and Technology, Makkah 41700, Saudi Arabia; 5 Florey Institute of Neuroscience and Mental Health, University of Melbourne, Melbourne, VIC 3052, Australia; cristiana.mattei@unimelb.edu.au; 6 St Vincent’s Institute of Medical Research, Fitzroy, VIC 3065, Australia; mlim@svi.edu.au; 7 Molecular Horizons, School of Medical, Indigenous and Health Sciences, University of Wollongong, Wollongong, NSW 2500, Australia

**Keywords:** autism, calcium, iPSC, neuron, development, differentiation, cortical

## Abstract

Autism Spectrum Disorder (ASD) is a neurodevelopmental condition that affects communication, social interaction, and behavior. Calcium (Ca^2+^) signaling dysregulation has been frequently highlighted in genetic studies as a contributing factor to aberrant developmental processes in ASD. Herein, we used ASD and control induced pluripotent stem cells (iPSCs) to investigate transcriptomic and functional Ca^2+^ dynamics at various stages of differentiation to cortical neurons. Idiopathic ASD and control iPSC lines underwent the dual SMAD inhibition differentiation protocol to direct their fate toward cortical neurons. Samples from multiple time points along the course of differentiation were processed for bulk RNA sequencing, spanning the following sequential stages: the iPSC stage, neural induction (NI) stage, neurosphere (NSP) stage, and differentiated cortical neuron (Diff) stage. Our transcriptomic analyses suggested that the numbers of Ca^2+^ signaling-relevant differentially expressed genes between ASD and control samples were higher in the iPSC and Diff stages. Accordingly, samples from the iPSC and Diff stages were processed for Ca^2+^ imaging studies. Results revealed that iPSC-stage ASD samples displayed elevated maximum Ca^2+^ levels in response to ATP compared to controls. By contrast, in the Diff stage, ASD neurons showed reduced maximum Ca^2+^ levels in response to ATP but increased maximum Ca^2+^ levels in response to KCl and DHPG relative to controls. Considering the distinct functional signaling contexts of these stimuli, this differential profile of receptor- and ionophore-mediated Ca^2+^ response suggests that aberrant calcium homeostasis underlies the pathophysiology of ASD neurons. Our data provides functional evidence for Ca^2+^ signaling dysregulation during neurogenesis in idiopathic ASD.

## 1. Introduction

Autism Spectrum Disorder (ASD) is a neurodevelopmental condition marked by impaired communication, social deficits, and repetitive behaviors [1]. Symptoms may present differently in different individuals with varying types and severity. Imaging and post-mortem studies of ASD document early aberrations of brain cytoarchitecture, specifically, enlarged brain size and alterations of dendritic spines [1,2]. Genetic analysis supports the heritability of ASD, as a significantly higher concordance rate has been found in homozygotic twins compared to heterozygotic siblings [3]. However, the genetic architecture of ASD seems to be heterogeneous and complex, whereby hundreds to thousands of genetic mutations have been linked to ASD, with no single mutation being responsible for more than 1–2% of cases [4]. Identified ASD genes are involved in diverse pathways that regulate brain development and synaptic formation and function [4]. Among the top ASD genetic candidates have been found several mutations in genes associated with calcium (Ca^2+^) signaling [5,6].

The initial recognition for Ca^2+^ dysregulation in ASD stems from Timothy syndrome, a monogenetic disorder with autistic features, caused by a de novo mutation in *CACNA1C*, which is a gene that encodes the alpha 1C subunit proteins of an L-type voltage-gated Ca^2+^ channel [7]. Cumulative studies of the idiopathic form of ASD increasingly implicate Ca^2+^ dysregulation in the pathogenesis of the disorder. Genetic studies identified a de novo copy number variant in the inositol triphosphate receptor (IP3R) gene in ASD patients [8]. In addition, functional studies using fibroblasts derived from both monogenetic and sporadic ASD patients showed a shared phenomenon of depressed Ca^2+^ release through IP3R [9,10]. In parallel, animal studies showed that mice with astrocyte-specific knockout of IP3R displayed autistic symptoms, mainly resulting from compromised Ca^2+^ signaling and deficiency in ATP release from astrocytes, which subsequently led to dysfunctional purinergic signaling in neurons [11]. Given the versatility and diversity of Ca^2+^ signaling functions, it has been proposed that Ca^2+^ signaling may represent a major hub where genetic abnormalities in ASD converge to produce their deleterious effect, but this requires investigation, especially in the context of human brain development.

The lack of access to living human brains and the limited applicability of animal studies to human brain regions responsible for higher functions are major obstacles to understanding cellular mechanisms that underlie brain disorders, including ASD. Induced pluripotent stem cells (iPSCs) offer a robust complementary tool to study complex human brain disorders in the context of development in vitro [12,13]. Several studies have examined idiopathic ASD and/or investigated ASD specific mutations using iPSC technologies and showed aberrant Ca^2+^ signaling [14,15,16,17,18,19,20]. DeRosa et al. (2018) [14] conducted a time course transcriptomic analysis on neurons derived from idiopathic ASD iPSCs spanning two time points during the process of differentiation to cortical neurons, including day 35 and day 135. Their results yielded Ca^2+^ signaling as one of the top pathways dysregulated in ASD, especially early during differentiation at day 35. This was further supported by Ca^2+^ imaging, which showed a significant decrease in the number of spontaneous Ca^2+^ transients in day 35 differentiated neurons [14]. Another study performing transcriptomic analyses across different stages of neuronal differentiation in ASD showed that the pathology starts as early as iPSCs start differentiating to neurons and that among the top significant signaling pathways implicated early during the differentiation is Ca^2+^ signaling [15]. Avazzadeh et al. (2019) [16] reported that ASD iPSC-derived cortical neurons harboring a heterozygous mutation in *NRXN1* alpha displayed altered Ca^2+^ release kinetic compared to controls, including higher frequency, longer duration, and bigger amplitude. They also performed transcriptomic analyses and found that among the top differentially expressed genes between ASD and control neurons are genes that encode voltage-gated calcium channels. Teles et al. (2022) [17] investigated Ca^2+^ homeostasis in iPSC-derived neurons harboring a heterozygous missense mutation in the *RELEN* gene and a de novo splice site variant in *CACNA1H*. ASD cells showed enhanced Ca^2+^ influx through Cav3.2 and displayed overactivation of mTORC1 signaling and impaired Reelin signaling. Several recent studies investigating specific ASD risk genes or variants also reported impaired Ca^2+^ signaling in neurons derived from iPSCs [18,19,20]. Altogether, these studies implicate aberrant Ca^2+^ signaling in the pathogenesis and pathophysiology of ASD early during neuronal differentiation.

Whilst many studies investigated Ca^2+^ signaling in ASD using iPSC models, the majority focused on studying specific ASD mutations or risk variants and characterizing spontaneous calcium transients. Herein, we sought to conduct stimulus response functional characterizations to investigate receptor-mediated Ca^2+^ dynamics in idiopathic ASD. We first performed transcriptomic analyses in samples from multiple stages along the course of differentiation, sequentially including the iPSC stage, neural induction stage (NI), neurosphere stage (NSP), and differentiated cortical neurons stage (Diff). Our data revealed that the numbers of Ca^2+^ signaling-relevant differentially expressed genes (DEGs) between the ASD and control groups were higher in samples from the iPSC and differentiated (Diff) stages. Accordingly, we performed our functional characterization of Ca^2+^ at the iPSC and Diff stages. Our findings indicated that iPSC-stage ASD samples displayed elevated maximum Ca^2+^ levels in response to ATP compared to controls. By contrast, differentiated ASD neurons exhibited reduced maximum Ca^2+^ levels in response to ATP stimulation but showed elevated maximum Ca^2+^ levels compared to control neurons following stimulation with KCl and (S)-3,5-dihydroxyphenylglycine (DHPG), a selective group I metabotropic glutamate receptor agonist. This may suggest that ASD neurons have higher excitability and/or mature at a faster rate than control neurons. These results provide complementary evidence for Ca^2+^ signaling dysregulation in ASD and support the use of the iPSC differentiation system as a robust model to study developmental disorders in vitro.

## 2. Materials and Methods

### 2.1. Derivation, Characterization, and Maintenance of Stem Cell Lines

Stem cells were utilized in accordance with the guidelines and regulations of King Faisal Specialist Hospital and Research Centre (KFSH&RC) and with the approval of the Office of Research Affairs Research Ethics Committee and Basic Research Committee (Project no: 2190006 and approval reference no: C380/345/41). Two ASD iPSC lines (SC119 and SC125) and two healthy iPSC controls (007 and FAM) were included in this study (Table 1). The two idiopathic ASD iPSC lines were generously provided by the University of Melbourne, Melbourne, Australia, under an approved and signed material transfer agreement. Healthy iPSC line 007 was kindly provided by Prof. Alice Pébay (UoM, Melbourne, Australia) [21,22], and iPSC line FAM was kindly provided by Dr. Reem Alhejilan (KFSH&RC, KSA, Riyadh, Saudi Arabia) [23]. Cells were cultured using the feeder free system on vitronectin-coated 60 mm dishes (Stem Cell Technologies, Vancouver, BC, Canada, CAT#: 07180), maintained in TeSR™-E8 defined medium as per the manufacturer’s instructions (Stem Cell Technologies, CAT#: 05990). Colonies were mechanically dissected on a weekly basis and replated on freshly coated plates to maintain their pluripotency, and they were regularly assessed for pluripotency by morphology and expression of OCT4. Cells were maintained at 37 °C under 5% CO_2_ and medium changes were performed daily.

Fibroblasts of ASD iPSC lines were originally obtained from Dr. Philip Schwartz (Children’s Hospital of Orange County Research Institute, Orange, CA, USA). ASD Fibroblasts were reprogrammed in the Dottori laboratory using the LONZA Transfection System (Lonza Group AG, Basel, Switzerland, CAT#: VP100) with non-viral episomal vectors provided by the Epi5 Episomal iPSC Reprogramming Kit (Life Technologies, Carlsbad, CA, USA, CAT#: A15960). The generated ASD iPSC lines were validated for pluripotency through the expression of key markers using the immunofluorescence assay (Figure S1). They were also assessed for their differentiation potential toward endoderm, mesoderm, and ectoderm derivates (Figure S2).

For endoderm differentiation, embryoid bodies (EBs) were generated from both ASD lines and harvested at day 14 for RT-PCR analysis. Total RNA was extracted using the PureLink RNA Kit according to the manufacturer’s instructions (Life Technologies, CAT#:12183025), and one microgram of RNA was used to synthesize first-strand cDNA using the Sensifast cDNA Synthesis Kit (Bioline, Cincinnati, OH, USA, CAT#; BIO-65054), according to the manufacturer’s instructions, under the following conditions: 25 °C for 10 min (primer annealing), 42 °C for 15 min (reverse transcription), 85 °C for 5 min (inactivation), and 4 °C hold. Each final reaction contained 1 µg cDNA in a volume of 20 µL. The GATA6 transcript—an endoderm marker—was amplified using the OneTaq RT/PCR Kit (BioLabs, Boston, MA, USA, CAT#: E5310S), following the manufacturer’s instructions, and visualized using a FluorChem E blot analyzer (ProteinSimple, San Jose, CA, USA). The following primers sequences were used: GATA6 (F: GCC TCA CTC CAC TCG TGT CT; R: TCA GAT CAG CCA CAC AAT ATG A) and BETA ACTIN (F: CAC CAC ACC TTC TAC AAT GAG C; R: TCG TAG ATG GGC ACA GTG TGG G).

For mesoderm differentiation, both ASD lines were differentiated toward cardiac specification. IPSCs were dissociated into single cells with TrypLE (Thermo Fisher Scientific, Waltham, MA, USA) and seeded onto plates coated with hESC-qualified Matrigel (Corning, Corning, NY, USA) at a density of 1 × 10^5^ cells/cm^2^ in TeSR-E8 medium supplemented with 10 µM Y-27632 (Tocris Bioscience, Bristol, UK). After 2 days, which is referred to as day 0, the medium was replaced with RPMI 1640 basal medium containing B-27 without insulin supplement (Thermo Fisher Scientific), growth factor-reduced Matrigel (1:60 dilution), and 10 µM CHIR99021 (Cayman Chemical, Ann Arbor, MI, USA). After 24 h, cells were initially treated with 5 ng/mL Activin A (Peprotech, Cranbury, NJ, USA) in RPMI 1640 basal medium containing B-27 without insulin supplement for 24 h. On day 2, the medium was changed to RPMI 1640 basal medium containing B-27 without insulin supplement and 5 µM IWP2 (Tocris Bioscience) for 72 h. Starting from day 5, cultures were maintained in RPMI 1640 supplemented with B-27 (Thermo Fisher Scientific) and 200 µg/mL L-ascorbic acid 2-phosphate sesquimagnesium salt hydrate (Sigma-Aldrich, St. Louis, MO, USA). On day 14, the samples were fixed for immunofluorescence analysis.

For ectoderm differentiation, both ASD iPSC lines were differentiated toward neuroectoderm specification as described below (see Section 2.2). Neurospheres (NSPs) at day 28 of differentiation were fixed for the immunofluorescence assay.

### 2.2. Differentiation of iPSCs

**Figure 1:**
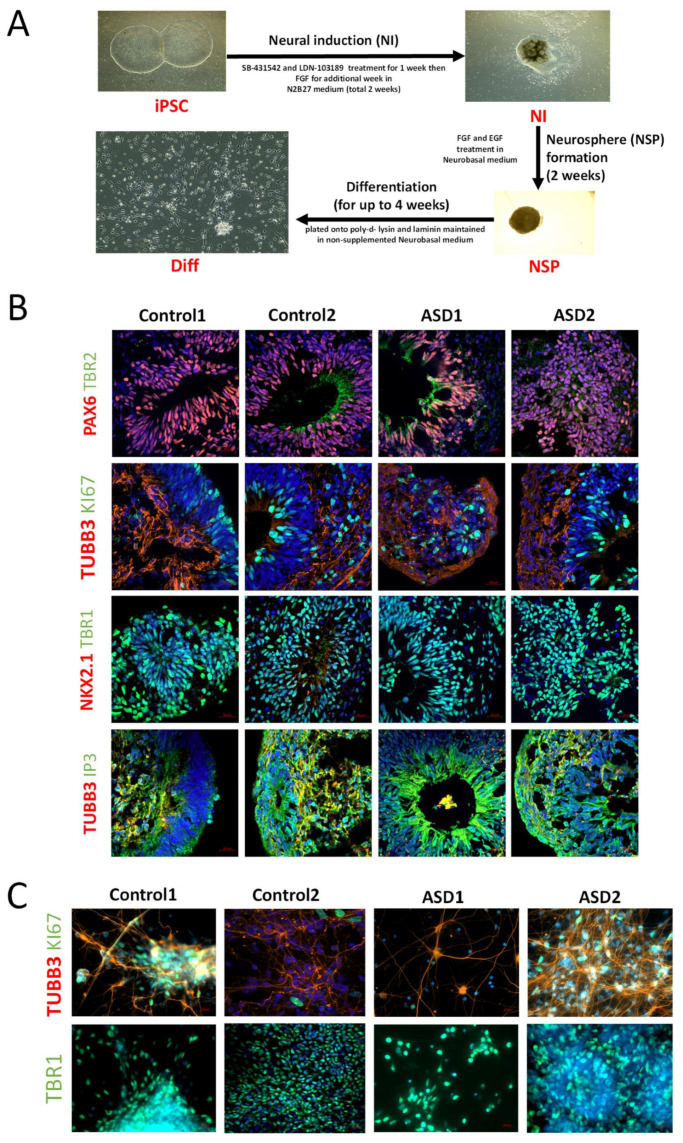
Immunofluorescence characterization of cortical neurons derived from ASD and control iPSCs. (**A**) Schematic of the dual SMAD inhibition protocol to generate cortical neurons from iPSCs. This protocol started with the neural induction stage (NI), which involved treatment of iPSCs with the small molecule SB431542 and LDN for 7 days, followed by treatment with FGF2 for another 7 days. Progenitors were then harvested and maintained in suspension in neurobasal medium supplemented with FGF2 and EGF for one week to form neurospheres (NSP stage). After two weeks, NSPs were plated onto poly-D-lysine and laminin-coated surfaces to terminally differentiate into neurons and maintained in culture for up to 4 weeks post-plating (Diff stage). (**B**) Immunofluorescence analyses of NSPs derived from iPSCs for each of cell lines used in this study: 007, FAM, SC119, and SC125. Cells within NSPs showed expression of neural progenitor marker PAX6, neuronal marker TUBB3, proliferative marker KI67, deep cortical layer marker TBR1, and inositol trisphosphate receptor (IP3). Scale bar = 20 µm. (**C**) Immunofluorescence analyses of 4-week-old differentiated neurons derived from iPSCs of cell lines used in this study: 007, FAM, SC119, and SC125. Differentiated neurons expressed neuronal marker TUBB3 and deep cortical layer marker TBR1. Scale bar = 20 µm.

For neural induction and differentiation of iPSC lines, the dual SMAD inhibition protocol was adopted as previously described by Denham and Dottori, with some slight modifications (Figure 1A) [24]. Briefly, iPSC lines were mechanically dissected into pieces approximately 0.5 mm in width and transferred onto laminin-coated organ culture plates in N2B27 medium containing 1:1 mix of neurobasal medium with DMEM/F12 medium, supplemented with 1% insulin transferrin selenium, 1% N2, 1% retinol-free B27, 0.3% glucose, 25 U/mL penicillin, and 25 μg/mL streptomycin (Thermo Fisher Scientific, CAT#: 11320033, A3582901, 17502-048, 12587-010, 51300-044, 25030-081, and 15070063, respectively). For induction of cortical neurons, the small molecule inhibitor SB431542 (10 μM, Tocris, CAT#: 1614) and LDN (100 ng/mL, Peprotech, CAT#6053) were added to the medium for the first 7 days, followed by the addition of FGF2 (20 ng/mL, Peprotech, CAT#: 130093841) for the remaining 7 days. Following 2 weeks of neural induction (NI), neural progenitors were mechanically harvested and cultured in suspension in neural basal medium (NBM) supplemented with FGF2 (20 ng/mL, Peprotech, CAT#: 130093841) and EGF (20 ng/mL, Peprotech, CAT#: AF-100-15) to promote NSP formation [25]. For terminal differentiation (Diff), NSPs were mechanically dissociated, seeded onto 25 mm coverslips coated with poly-D-lysine (SIGMA Aldrich, St. Louis, MO, USA, CAT#: P1024) and laminin (Life Technologies, CAT#: 23017015), and maintained in NBM medium without supplements for up to 4 weeks. Cells were maintained at 37 °C in 5% CO_2_ incubators and the medium was changed every second day.

### 2.3. Immunofluorescence

Cell monolayers (samples from iPSC, NI, and Diff) and NSPs were fixed in 4% PFA for 20 min at 4 °C and then washed briefly in PBS. NSPs were embedded in Tissue-Tek OCT compound (Labtek, Grand Rapids, MI, USA), cut in 15 μm slices on a cryostat, and sections were placed on superfrost slides. After washing in PBS, sections or culture dishes were permeabilized in 0.1% Triton-X-100 in PBS (PBT) for 5 min and then blocked in 10% fetal calf serum in PBT for 60 min at room temperature. Samples were then incubated with primary antibody (diluted in the block buffer) overnight at 4 °C. The following primary antibodies were used: OCT4A (R&D, Minneapolis, MN, USA, CAT# MAB17591), SOX2 (R&D, CAT# MAB2018), NANOG (R&D, CAT#:AF1997), TRA160(R) (R&D, CAT#MAB4770), ACTC1 (Sigma-Aldrich), PAX6 (Santa Cruz, Dallas, TX, USA, CAT#: sc-81649), TBR2 (Abcam, Cambridge, UK, CAT#: ab23345), TBR1 (Abcam, CAT#: ab31940), CTIP2 (Abcam, CAT#: ab18465), SATP2 (Abcam, CAT#: ab51502), TUBB3 (Millipore, Burlington, MA, USA, CAT#: MAB1637), KI67 (Abcam, CAT#: ab15580), NKX2.1 (Abcam, CAT#: ab72876), and IP3R (Abcam, CAT#: ab5804). Following three 5 min washes in PBT, ALEXA-fluor secondary antibodies (Life Technologies/Invitrogen, Carlsbad, CA, USA) (1:1000 diluted in the block buffer) were applied for 1 h at room temperature. All samples were counterstained with 49,6-diamidino-2-phenylindole (Dapi; 1 μg/mL, SIGMA-Aldrich). Samples were then mounted onto glass slides with Mowiol aqueous mountant, followed by viewing and image capturing under Zeiss spinning wheel confocal microscope Axio Observer 7 (Carl Zeiss Microscopy GmbH, Jena, Germany) using ZEN imaging software version 3.4.

### 2.4. RNA Isolation and Transcriptome Analysis

RNA was isolated from samples using the Qiagen RNeasy Mini Kit, as per the manufacturer’s instructions. In our study, we used two independent iPSC lines reprogrammed from two ASD donors and two independent iPSC lines reprogrammed from two control donors. Each line represented one clone per donor; thus, the biological replicates were donor-derived iPSC lines (*n* = 2 per group). All four lines underwent the dual SMAD inhibition differentiation protocol on three independent occasions. At each differentiation run, samples were collected at four developmental stages: (i) the iPSC stage (at least three colonies per dish, 3 dishes per line), (ii) the NI stage (day 14 rosettes derived from at least three colonies, 3 dishes per line), (iii) the NSP stage (2-week-old NSPs, 12–18 NSPs per line), and (iv) the Diff stage (neurons differentiated from ~12 NSPs plated into 3 dishes per line). For RNA-seq, RNA was extracted independently from each replicate dish (or NSP batch) per line and time point. Following extraction, RNA samples from replicate dishes within the same line and time point were pooled prior to library preparation. Thus, the unit of biological replication was the iPSC line (one clone per donor), while technical variability between dishes was averaged by pooling post-extraction.

For each of the four iPSC lines (two ASD and two control lines), we collected RNA samples at four time points along the differentiation trajectory: iPSC, 2-week NI, 2-week NSP, and 4-week Diff. This resulted in a total of 16 RNA samples, representing four independent biological replicates (iPSC lines) per time point. The concentration and overall quality of RNA samples were assessed using a Nanodrop ND-100 spectrophotometer and Agilent 2100 Bioanalyzer, with all samples showing an RNA integrity number (RIN) greater than 8 (Figure S7).

RNA sequencing and bioinformatics analysis were outsourced to BGI Genomics (Hong Kong) using the DNBSEQ platform (MGI Tech Co., Ltd., Shenzhen, China), averagely generating about 6.62G Gb bases per sample. The sequencing data were filtered using SOAPnuke. All of the samples had greater than 80% clean reads after filtering out reads of low quality (base quality ≤ 15), reads with adaptor sequences, and reads with high levels of unknown base N. The clean reads were mapped to human genome reference hg19 GRCh37 using HISAT. The average total mapping ratio with the reference genome was 95.92%, and the average total mapping ratio with genes was 74.28%. A total of 18,119 genes were identified.

For gene expression analysis, the clean reads were mapped to reference transcripts using Bowtie2. Analysis was carried out to compare ASD and control samples at each of the 4 stages of differentiation, including iPSC, NI, NSP and Diff. Gene expression level was determined using RSEM (v1.2.28), which provided read count, FPKM, and TPM values. Differential expression gene (DEG) analysis was carried out through DESeq2 with a Q value of ≤0.05. The resulting DEG analysis findings were visualized in a heatmap using pheatmap. To gain insight into changes in the phenotype, Gene Ontology (GO) enrichment analysis and Kyoto Encyclopedia of Genes and Genomes (KEGG) enrichment analysis were performed using the phyper function in R software (R Foundation for Statistical Computing, Vienna, Austria). The significance levels of both terms and pathways were subjected to correction by Q values, applying a stringent threshold (Q value ≤ 0.05).

### 2.5. Calcium Imaging

For calcium imaging, we first recorded timelapse images from iPSC colonies. For these experiments, three colonies per line were plated on coverslips, and recordings were obtained from at least three coverslips for each line and condition (ATP and KCl stimulation). This procedure was repeated at three independent times. We then recorded timelapse images from differentiated neurons. Two-week-old NSPs (3–6 per coverslip) were plated on three coverslips per line, and recordings were obtained at both 1 week and 4 weeks post-differentiation. For each time point and condition (ATP, KCl, and DHPG stimulation), at least three coverslips were analyzed per line. This workflow was also repeated across three independent differentiation runs. In both cases, the unit of biological replication was the iPSC line, while multiple coverslips and cells within each line provided technical replication.

IPSC colonies and 1-week-old and 4-week-old cultured differentiated neurons were loaded with Ca^2+^ indicator Fluo-4 AM (Thermo Fisher Scientific) in freshly prepared regular Krebs-HEPES buffer with a PH of 7.4 for a duration of 30 min and then were processed for imaging, as previously described [26]. Krebs-HEPES buffer contained 120 mM NaCl, 1.3 mM CaCl_2_, 1.2 mM MgSO_4_, 4.8 mM KCl, 1.2 mM KH_2_PO_4_, 25 mM HEPES, and 0.1% BSA. All salts, HEPES, and BSA were prepared from respective powders dissolved in ultrapure (deionized-distilled) water produced by the Milli-Q^®^ Integral Water Purification System.

Image acquisition and analyses were performed using Zeiss LSM META 510 laser scanning confocal microscope and CLSM software version 3.2. Serial images were acquired at approximately 1 s intervals. A baseline recording was first taken, then the stimulus was delivered at second 20, whereby cells were either stimulated with 25 mM KCl, 100 µM ATP (Tocris, CAT#3245), or 200 µM DHPG (SIGMA Aldrich, CAT#: D368), followed by 4 μM ionomycin at second 200 and EGTA at second 400. Imaging of cells was performed at 37 °C.

For analysis, we performed background subtraction, then we calculated ratio changes by normalizing intensity changes to the averaged intensity of the first three images before delivering the stimulus. Further analysis was performed to compare ASD and control neurons in terms of rate of rise, maximum receptor mediated response, and maximum ionomycin response. For both statistical analysis and visualization, data from the two ASD cell lines were combined into a single ASD group, and data from the two control cell lines were combined into a single control group for each condition: iPSC, 1-week Diff, and 4-week Diff. Statistical analysis was performed to compare between ASD and control datasets using the two-tailed Mann–Whitney U test in GraphPad Prism version 5.04 (GraphPad Software, San Diego, CA, USA).

## 3. Results

### 3.1. Derivation of Cortical Neurons from ASD and Control iPSC Lines

Two ASD iPSC lines were generated from fibroblasts originally provided by Dr. Philip Schwartz (Children’s Hospital of Orange County Research Institute, California) (Table 1). Cells were validated for pluripotency through the expression of key markers, including OCT4, SOX2, TRA1-60, and NANOG (Figure S1). Their differentiation potential was further confirmed via EB formation or directed in vitro differentiation, demonstrating their ability to generate derivatives of all three germ layers (Figure S2). We employed two male control cell lines for a direct comparison with patient iPSCs.

Cortical neurons play a pivotal role in the brain’s higher functions, such as sensory processing, communication, and social behavior, the very functions affected in ASD [27]. We therefore sought to specifically differentiate ASD and control iPSC lines to cortical-like neuronal populations. To achieve this, we adopted the dual SMAD inhibition protocol, which is a well-established method and a well-characterized protocol in the Dottori laboratory to obtain cortical-like neurons [24,25,28]. This protocol involves three sequential steps to convert iPSCs into relatively mature cortical neurons, including NI, NSP, and Diff (Figure 1A).

NI was the first step, whereby iPSCs were treated with the small molecule SB431542 and LDN for 7 days, followed by treatment with FGF2 for another 7 days. Colonies at the end of NI showed rosette-like structures representative of the neuroepithelia of the neural tube and immunostaining analyses revealed the expression of neural progenitor marker SOX2 and dorsal telencephalic neuroepithelial marker PAX6 (Figure S3). Transcriptomic data revealed that samples from the NI stage had higher expression of dorsal telencephalic marker *PAX6* and forebrain marker *FOXG1* compared to the iPSC stage (Figure S4A,B).

NSP formation was the second step, whereby progenitors were harvested and maintained in suspension in NBM medium supplemented with FGF2 and EGF for two weeks to form NSPs (Figure 1A). This stage also coincided with progenitor differentiation toward deep layer cortical subpopulations. Immunostaining revealed strong expression of PAX6 and the deep layer cortical marker TBR1, suggesting a progression toward post-mitotic cortical neuron fate, with limited the expansion of intermediate progenitors, as indicated by low TBR2 expression. Cells also expressed TUBB3, confirming neuronal identity, and a subset was positive for Ki67, reflecting ongoing proliferation within the culture (Figure 1B). While, as expected, minimal to no expression was observed for ventral telencephalic marker NKX2.1 and intermediate and upper layer markers CTIP2 and BRN1. This suggested that 2-week-old NSPs were mainly enriched with neuronal populations of dorsal telencephalic identity, specifically the deep layer cortical neuronal populations (Figure 1B).

Diff was the third step, whereby 2-week-old NSPs were mechanically dissociated and plated onto poly-D-lysine and laminin coated surface to terminally differentiate neural progenitors to post-mitotic neurons (Figure 1A). Immunostaining analyses of neuronal cultures within the fourth week post-differentiation showed the expression of neuronal markers TUBB3 and TBR1, suggesting enrichment of deep cortical neuronal populations in Diff cultures. Additionally, the presence of Ki67-positive cells suggested that a subset of the culture remained proliferative at this stage (Figure 1C). Complementing these results, our transcriptomic analyses also showed higher levels of *TUBB3* in NSP and Diff samples compared to iPSC samples (Figure S4C).

Building on these observations, transcriptomic analyses further revealed the altered expression of genes associated with neuronal differentiation in ASD-derived Diff samples relative to controls, including upregulation of the pyramidal neuron marker *EMX1* and the layer 6 cortical neuron marker *TBR1* (Figure S5). These molecular changes may reflect underlying alterations in corticogenesis associated with ASD. While these transcriptomic data provide valuable insights into stage-specific gene expression patterns, the limited sample size constrained our ability to perform formal statistical analyses. These findings suggest potential deviations in the neuronal differentiation trajectory of ASD-derived neurons.

### 3.2. Transcriptomic Evidence for Ca^2+^ Dysregulation in ASD

**Figure 2:**
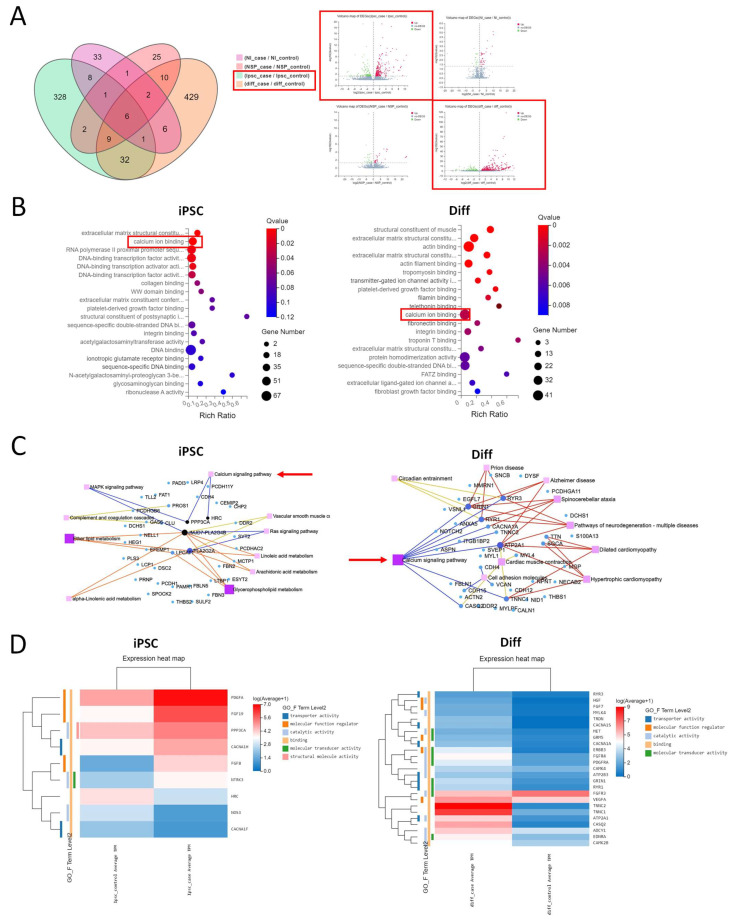
Transcriptomic profiles of ASD and control samples at 4 stages along the course of differentiation, including the iPSC stage, neural induction (NI) stage, neurosphere (NSP) stage, and 4-week differentiation (Diff) stage. (**A**) The Venn diagram (**left**) shows the distribution of differentially expressed genes (DEGs) between ASD and control samples at each of the 4 stages: iPSC, NI, NSP, and Diff. The volcano plot (**right**) shows DEGs of ASD vs. control samples at the iPSC stage (**top left**), NI stage (**top right**), NSP stage (**bottom left**), and Diff stage (**bottom right**). The *X*-axis represents the fold change of the difference after conversion to log2, and the *Y*-axis represents the significance value after conversion to −log10. Red represents upregulated DEGs, green represents downregulated DEGs, and gray dots represent genes that are non-DEGs. (**B**) The DEGs of ASD vs. control samples at the iPSC (**left**) and Diff (**right**) stages were analyzed for GO molecular function and presented as bubble chart. Bubble size reflects the number of enriched genes and color represents statistical significance. (**C**) KEGG network diagram of DEGs in the calcium signaling pathway at the iPSC stage (**left**) and Diff stage (**right**). The large red arrows highlight the calcium signaling pathway, indicating its prominent localization in the dataset. (**D**) Heatmaps showing DEGs of ASD vs. control samples in the calcium signaling pathway at the iPSC stage (**left**) and Diff stage (**right**).

Ca^2+^ signaling has been identified by previous transcriptomic studies using iPSC models as a major hub in ASD pathophysiology [15]. Accordingly, we sought to conduct a transcriptomic profile of samples collected from different time points along the course of differentiation to identify the developmental stages affected by dysregulation of Ca^2+^ signaling. To achieve this, we performed bulk RNA sequencing analysis of each cell line (2 ASD and 2 control lines) at four sequential stages of the differentiation—iPSC, 2-week NI, 2-week NSPs, and 4-week Diff. The analysis was carried out to compare the gene expression profiles of ASD and control samples at each of the mentioned 4 stages of differentiation. Using a Q value of <0.05, our data revealed that the number of DEGs was highest in the Diff stage, with a total number of DEGs corresponding to 495, followed by the iPSC stage, corresponding to 387, while the numbers of DEGs were lower in the NI and NSP stages, corresponding to 58 and 56, respectively, with a general trend of more upregulated than downregulated genes (Figure 2A). Tables S1 and S2 illustrate the list of significant DEGs at the iPSC and Diff stages.

DEGs in the iPSC and Diff stages were then subjected to functional classification and GO term enrichment analysis. Results suggested that binding was the most enriched GO term under Molecular Function, corresponding to 360 DEGs in the iPSC stage and 441 DEGs in the Diff stage. Further analyses revealed that, in the iPSC stage, 39 DEGs were annotated as Ca^2+^ ion binding (Q value = 0.00011) and, in the Diff stage, 39 DEGs were also annotated as Ca^2+^ ion binding (Q value = 0.002) (Figure 2B). We also subjected DEGs in the iPSC and Diff stages to KEGG pathway annotation classification. Our results revealed that 25 DEGs in the Diff stage were classified under the term Calcium Signaling Pathway, with a Q value of 0.00000014. On the other hand, 9 DEGs in the iPSC stage were identified under the term Calcium Signaling Pathway but did not reach statistical significance (Q value = 0.79). The KEGG network map and expression heatmap for these DEGs identified under the term Calcium Signaling Pathway in the iPSC and Diff stages are shown in Figure 2C,D. Among the DEGs in the Diff stage, we found upregulated genes that are well recognized for their integral role in maintaining resting Ca^2+^ and facilitating different Ca^2+^ signals, some of which were already reported in previous studies. These include ryanodine receptors (*RYR1/3*), calcium voltage-gated channel subunit alpha1 S (*CACNAS*), calcium/calmodulin dependent protein kinase IV (*CAMK4*), glutamate metabotropic receptor 5 (*GRM5*), ATPase plasma membrane Ca2+ transporting 3 (*ATP2B3*), glutamate ionotropic receptor NMDA type subunit 1 (*GRIN1*), and calcium/calmodulin dependent protein kinase II beta (*CAMK2B*). Given the crucial role of Ca^2+^ signaling in regulating distinctive processes during different stages of neurodevelopment, we were prompted to validate these findings and investigate the biological implication of such disruptions.

### 3.3. ASD iPSCs Display Altered Ca^2+^ Homeostasis and Dynamics

**Figure 3:**
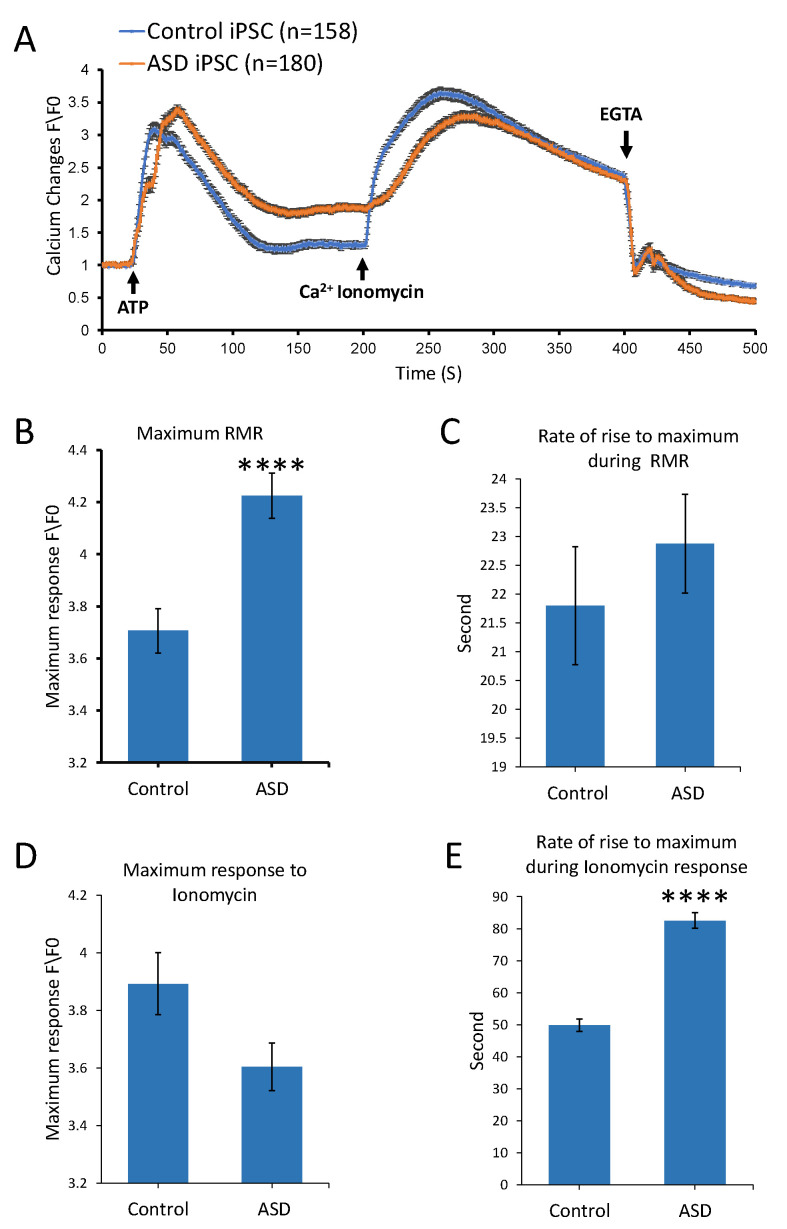
Pharmacological characterization of calcium (Ca^2+^) dynamics in ASD and control samples at the iPSC stage. (**A**) The graph shows average intensity changes of Ca^2+^ indicator Fluo-4 AM in response to ATP in ASD and control iPSCs, two cell lines each. A baseline recording was first taken, then the stimulus, 100 µM ATP, was delivered at second 20, followed by 4 µM ionomycin at second 200 and EGTA at second 400. Experiments were conducted using three independent differentiation setups for each cell line, with each setup including three technical repeats. Data shown in (**A**) represent the average results from two cell lines across three independent experiments, expressed as normalized fluorescence intensity ratio relative to the averaged three images obtained prior to the addition of stimulus. (**B**–**E**) graphs show comparisons between ASD and control iPSCs in (**B**) maximum receptor mediated Ca^2+^ response (RMR) to ATP, (**C**) rate of rise to maximum during RMR, (**D**) maximum response to ionomycin, and (**E**) rate of rise to maximum during response to ionomycin. Bars represent the average results from two cell lines across three independent experiments, with error bars indicating standard error of the mean. Statistical analysis was performed using a the two-tailed Mann–Whitney U test. Statistical significance is indicated by asterisks **** *p*  < 0.0001.

We performed Ca^2+^ imaging studies of ASD and healthy control samples at multiple stages of differentiation, starting with the pluripotency stage. To assess the impact of dysregulation in the Ca^2+^ signaling-related transcriptome on ASD iPSCs, ATP was used in these studies to stimulate purinergic receptors and induce Ca^2+^ release from internal stores. A similar approach and settings to previously published studies from our group were adopted [29]. Briefly, Fluo-4-loaded iPSC colonies from the ASD and control groups were processed for time lapse recordings for a duration of 500 s. A baseline recording was first taken, then the stimulus, 100 µM ATP, was delivered at second 20, followed by 4 µM ionomycin at second 200 and EGTA at second 400 (Figure 3A). Both ASD and control iPSC lines showed homogeneous responses to ATP. Interestingly, ASD iPSCs had higher levels of intracellular Ca^2+^ in response to ATP, reaching an average maximum change of 4.23 ± 0.085 fold (normalized fold increase relative to baseline) compared to control iPSCs, which reached an average maximum change of 3.71 ± 0.085 fold (*p* value ≤ 0.0001, *n* = 3) (Figure 3B). On the other hand, ASD iPSCs had slightly lower levels in response to ionomycin, reaching a maximum change of 3.60 ± 0.081 fold compared to control iPSCs, which reached a maximum change of 3.89 ± 0.10 fold (not statistically significant, *n* = 3) (Figure 3D). The rise in intracellular Ca^2+^ to reach the maximum levels in ASD iPSCs was significantly slower, taking an average time of around 82.59 ± 2.45 s compared to controls, which corresponded to 49.87 ± 1.91 s (*p* value ≤ 0.0001, *n* = 3) (Figure 3E). These results confirmed the findings of our transcriptomic analyses, that there are differences in Ca^2+^ homeostasis and dynamics between ASD and control samples at the iPSC stage.

### 3.4. Ca^2+^ Dynamics Are Affected in ASD Early Cortical Neurons

Our transcriptomic data indicated that the highest number of Ca^2+^ signaling-relevant DEGs was in the Diff neuronal stage. Therefore, we sought to investigate functional Ca^2+^ differences at the Diff neuronal stage, and we selected to perform these studies at two time points post-differentiation, early at 1-week Diff and late at 4-week Diff. For studies conducted at the 1-week Diff time point, we characterized the dynamics of intracellular Ca^2+^ upon stimulation with either KCl, to probe the release of Ca^2+^ through activation of voltage-gated Ca^2+^ channels, or ATP, to probe the release of Ca^2+^ through activation of the purinergic receptor and downstream IP_3_ pathway signaling.

**Figure 4:**
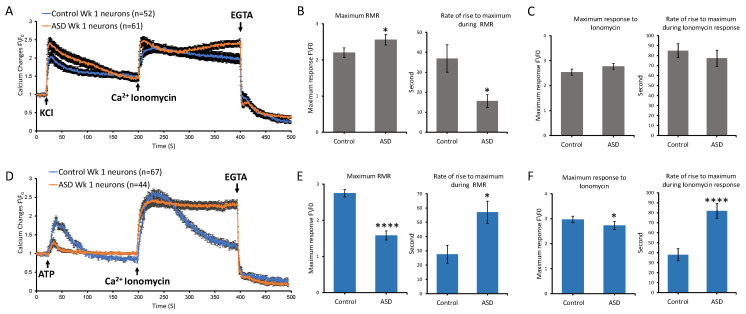
Pharmacological characterization of calcium (Ca^2+^) dynamics in ASD and control 1-week-old neurons at the Diff stage. Graphs show average intensity changes of Ca^2+^ indicator Fluo-4 AM in response to KCl in (**A**) and ATP in (**D**) in 1-week Diff neurons derived from ASD and control iPSCs. A baseline recording was first, taken then the stimulus, either 25 mM KCl or 100 µM ATP, was delivered at second 20, followed by 4 µM ionomycin at second 200 and EGTA at second 400. Experiments were conducted using three independent differentiation setups for each cell line, with each setup including three technical repeats. Data shown in (**A**,**D**) represent the average results from two cell lines across three independent experiments, expressed as normalized fluorescence intensity ratio relative to the averaged three images obtained prior to the addition of stimulus. (**B**,**C**,**E**,**F**) graphs show comparisons between ASD and control 1-week Diff neurons in (**B**,**E**) maximum receptor mediated Ca^2+^ response (RMR) and rate of rise to maximum during RMR and (**C**,**F**) maximum response to ionomycin and rate of rise to maximum during response to ionomycin. Bars represent the average results from two cell lines across three independent experiments, with error bars indicating standard error of the mean. Statistical analysis was performed using the two-tailed Mann–Whitney U test. Statistical significance is indicated by asterisks: * *p* < 0.05, and **** *p*  < 0.0001.

Accordingly, we firstly sought to determine if there were differences between the ASD and control groups in Ca^2+^ dynamics during the early stage of differentiation upon stimulation with KCl. Our results indicated that the percentage of cells that had doubled their response to KCl was slightly higher for the ASD group, corresponding to 57.37 ± 6.33%, compared to 42.30 ± 6.85% for the control group. Data also showed that the average maximum response to KCl in ASD neurons was significantly higher than in controls, corresponding to an average intensity of 2.55 ± 0.14, compared to 2.20 ± 0.13 in controls (*p* value ≤ 0.05, *n* = 3) (Figure 4A–C). The rate of rise to reach the maximum was significantly faster in ASD neurons, corresponding to 15.57 ± 6.88 s, compared to 36.88 ± 3.21 s in the control group (*p* value ≤ 0.05, *n* = 3), overall suggesting that ASD neurons may have higher excitability than controls early (week 1) in the differentiation stage (Figure 4A–C). No significant differences were identified in terms of subsequent global Ca^2+^ rise in response to ionomycin in these experiments.

We then assessed whether the kinetics of Ca^2+^ release would be different between ASD 1-week neurons and controls upon activation of purinergic receptors by ATP. Our data showed that the percentage of ASD neurons that doubled their Ca^2+^ response was 13.63 ± 5.233%, which was lower compared to 85 ± 4.35% in controls. In addition, the average maximum values during ATP-mediated response in ASD neurons was lower, corresponding to 1.58 ± 0.12 (*p* value ≤ 0.0001, *n* = 3), and slower to reach the maximum, with an average duration of 57 ± 7.78 s compared to controls, which reached an average maximum of about 2.74 ± 0.10 s, with an average duration of 27.62 ± 6.30 s (*p* value ≤ 0.01, *n* = 3) (Figure 4D–F). The average maximum Ca^2+^ response to ionomycin was comparable between the two groups, although slightly less in the ASD group (ASD = 2.73 ± 0.16 fold, control = 2.97 ± 0.13 fold, *p* value ≤ 0.05, *n* = 3), with a significant longer duration to reach the maximum (ASD = 81.69 ± 7.52 s, control = 37.85 ± 6.10 s, *p* value ≤ 0.0001, *n* = 3) compared to the control group (Figure 4D–F). The response demonstrated by ASD and control neurons at the NI stage was different than at the iPSC stage, which was expected given that different machinery is operating to regulate and maintain Ca^2+^ according to different cellular context across different cell types. ATP and downstream purinergic signaling play an important role during neurogenesis and maintenance of neural stem cells. This was consistent with our observation that a homogeneous response to ATP was observed for both ASD and control samples at the iPSC stage. By contrast, in 1-week-old neurons, only a small percentage of ASD neurons responded to ATP compared to controls.

### 3.5. Ca^2+^ Dynamics Are Disrupted in ASD Cortical Neurons at Later Stage of Differentiation

We next sought to assess whether phenotypic differences in Ca^2+^ dynamics between ASD and control samples that were observed during the early phase of differentiation were also maintained at a later and more mature stage of differentiation, specifically week 4. In addition to KCl and ATP, we also used DHPG to assess the dynamics of Ca^2+^ upon activation of metabotropic glutamate receptors (mGLURs) given that neurons at this stage express mGLURs and are mature enough to respond to DHPG, unlike 1-week-old neurons, which showed minimal to no response.

**Figure 5:**
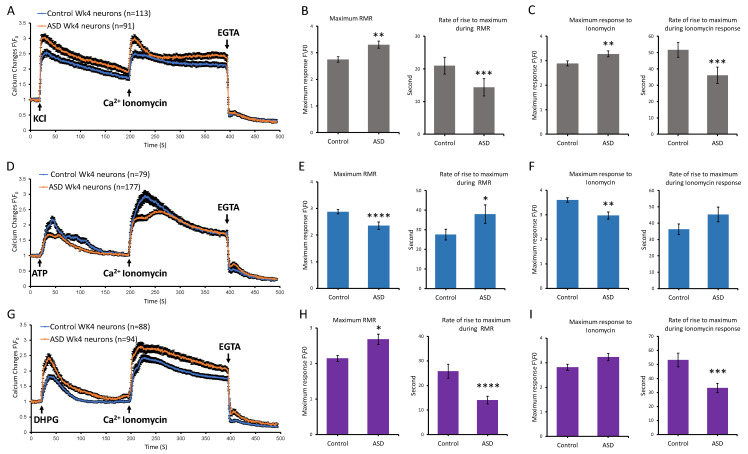
Pharmacological characterization of calcium (Ca^2+^) dynamics in ASD and control 4-week-old neurons at the Diff stage. Graphs show average intensity changes of Ca^2+^ indicator Fluo-4 AM in response to KCl in (**A**), ATP in (**D**), and DHPG in (**G**) in 4-week Diff neurons derived from ASD and control iPSCs. A baseline recording was first taken, then the stimulus, either 25 mM KCl, 100 µM ATP, or 200 µM DHPG, was delivered at second 20, followed by 4 µM ionomycin at second 200 and EGTA at second 400. Experiments were conducted using three independent differentiation setups for each cell line, with each setup including three technical repeats. Data shown in (**A**,**D**,**G**) represent the average results from two cell lines across three independent experiments, expressed as normalized fluorescence intensity ratio relative to the averaged three images obtained prior to the addition of stimulus. (**B**,**C**,**E**,**F**,**H**,**I**) graphs show comparisons between ASD and control 1-week Diff neurons in (**B**,**E**,**H**) maximum receptor mediated Ca^2+^ response (RMR) and rate of rise to maximum during RMR and (**C**,**F**,**I**) maximum response to ionomycin and rate of rise to maximum during response to ionomycin. Bars represent the average results from two cell lines across three independent experiments, with error bars indicating standard error of the mean. Statistical analysis was performed using the two-tailed Mann–Whitney U test. Statistical significance is indicated by asterisks: * *p*  < 0.05, ** *p* < 0.005, *** *p* < 0.0005, and **** *p* < 0.0001.

Our data showed that the differential profile of KCl-induced Ca^2+^ dynamics in 4-week-old neurons between ASD and control samples was comparable to the 1-week-old neurons, whereby ASD neurons showed a higher average maximum response (ASD = 3.30 ± 0.13, control = 2.75 ± 0.10, *p* value ≤ 0.001, *n* = 3) and significantly shorter duration (ASD = 14.38 s, control = 20.97 s, *p* value ≤ 0.0005, *n* = 3) to reach the maximum compared to controls (Figure 5A–C). The percentage of cells doubling their response relative to the resting level was slightly higher in ASD neurons (85.71% ± 3.66) compared to controls (75.22% ± 4.061). Compared to 1-week-old neurons, the response of 4-week-old neurons in both groups (ASD and control) was higher, suggesting that neurons at week 4 had increased excitability compared to 1-week Diff neurons. Overall, ASD neurons had increased responses to KCl at both 1-week and 4-week Diff time points compared to controls, which suggested that ASD neurons are more excitable (Figure 5A–C). Furthermore, differences in global Ca^2+^ dynamics upon the addition of ionomycin was significantly higher in ASD neurons, whereby ASD 4-week-old neurons showed a higher average maximum response (ASD = 3.28 ± 0.12 and control = 2.89 ± 0.10, *p* value ≤ 0.005, *n* = 3) and faster duration to reach the maximum (ASD = 36.16 ± 5.01 and control = 51.64 ± 4.46, *p* value ≤ 0.001, *n* = 3) compared to controls (Figure 5A–C).

We next assessed the dynamics of Ca^2+^ in ASD neurons upon stimulation with ATP and downstream purinergic signaling. Similar profiles were observed at 1 and 4 weeks, whereby ASD 4-week-old neurons showed a lower average maximum Ca^2+^ response (ASD = 2.35 ± 0.14 fold, Control = 2.88 ± 0.08 fold, *p* value ≤ 0.0001, *n* = 3) with a longer duration to reach the maximum (ASD = 38 ± 4.70 s, control = 27.53 ± 2.75 s, *p* value ≤ 0.05, *n* = 3) compared to controls (Figure 5D–F). The percentage of cells doubling their response to ATP was lower (58.19 ± 3.71%) in ASD 4-week-old neurons compared to controls (90.78 ± 3.32%). The profile of Ca^2+^ rise during the response to ionomycin in these experiments was also significant in ASD neurons compared to controls, where ASD neurons showed a lower average maximum (ASD = 2.97 ± 0.14, control = 3.60 ± 0.08, *p* value ≤ 0.005, *n* = 3) and longer duration (ASD = 45.35 ± 4.52, control = 36.27 ± 3.24, not statistically significant, *n* = 3) to reach the maximum compared to controls (Figure 5D–F).

We next assessed whether there would be differences between ASD and control neurons in the kinetics of Ca^2+^ release upon stimulation of mGLURs with DHPG. Given that our transcriptomic analyses suggested higher expression of mGLURs in ASD neurons compared to controls, we expected that the rise in Ca^2+^ in response to activation of mGLURs would be higher in ASD neurons compared to controls. Indeed, ASD 4-week-old neurons showed a higher average maximum Ca^2+^ response (ASD = 2.68 ± 0.14, control = 2.14 ± 0.08, *p* value ≤ 0.05, *n* = 3) with a shorter duration to reach the maximum (ASD = 14.07 ± 1.60 s, control = 25.86 ± 0.12 s, *p* value ≤ 0.0001, *n* = 3) compared to controls (Figure 5G–I). The percentage of cells doubling their response to DHPG was higher (59.57 ± 5.06%) in ASD 4-week-old neurons compared to controls (51.13 ± 5.32%). The response to ionomycin was also similarly different in ASD neurons compared to control neurons, whereby ASD neurons showed a higher average maximum (ASD = 3.22 ± 0.15, control = 2.81 ± 0.12, not statistically significant, *n* = 3) and a shorter duration to reach the maximum (ASD = 33.31 ± 3.40 s, control = 53.15 ± 4.94 s, *p* value ≤ 0.0005, *n* = 3), overall suggesting altered global intracellular Ca^2+^ dynamics in ASD (Figure 5G–I).

## 4. Discussion

This study investigated receptor-mediated Ca^2+^ release of idiopathic ASD iPSC-derived cortical neurons at different stages along their differentiation, including the iPSC, NI, NSP, and Diff stages. Our transcriptomic analyses showed that the numbers of Ca^2+^ signaling-relevant DEGs between the ASD and control groups were higher in samples from the iPSC and Diff stages, with the majority of DEGs being upregulated rather than downregulated. Accordingly, Ca^2+^ imaging studies were carried out in samples from the iPSC and Diff stages spanning two time points at the Diff stage, including 1-week and 4-week Diff neurons. Our characterizations included testing receptor-mediated Ca^2+^ release upon activation of purinergic receptors, voltage-gated calcium channels, and mGLURs receptors, using ATP, KCl, and DHPG, respectively. Our results suggested that ASD samples had altered Ca^2+^ transient kinetics in response to these stimuli compared to controls. At the iPSC stage, ASD samples displayed elevated maximum Ca^2+^ levels in response to ATP compared to controls. By contrast, ASD neurons from 1- and 4-week time points at the Diff stage exhibited notably reduced maximum Ca^2+^ responses to ATP and higher maximum responses to KCl and DHPG in comparison to controls. Our functional data showed that ionomycin-releasable stores were also affected differently in control versus ASD cells, pointing to multiple alterations in Ca^2+^ signaling, including the L-type Ca^2+^ channels (CACNA1S), which affects calcium influx magnitude and kinetics, SERCA pump (ATP2A1), which controls calcium clearance and store refiling, SERCA regulation (SLN) which fine-tunes calcium reuptake kinetics, and ryanodine receptors (RYR1 and RYI3), which control calcium release from internal stores and the calcium storage protein calsequestrin (CASQ2). Taken together, these results indicate aberrant calcium homeostasis as a result of multiple effects on calcium transients.

While our findings support the notion of early Ca^2+^ dynamics alteration during neurogenesis in ASD, it is important to acknowledge that the relatively small number of iPSC lines analyzed (two idiopathic ASD lines and two controls), along with the use of a single clone per line, limits the generalizability of our conclusions. Validating these findings across multiple clones and added individuals will be essential to account for inter-clonal variability and to capture the heterogeneity of ASD more accurately. In this context, the observed differences in the distribution and number of neuronal cells across samples may reflect primary differences in the differentiation process among lines, which could underlie subsequent genetic alterations due to the heterogeneous nature of the derived neurons. Line variation is a recognized limitation in the use of stem cells, both embryonic stem (hES) cell lines and iPSC lines [30,31,32,33,34,35]. The field has developed various strategies to address it, including reference cell lines and improved characterization methods to account for this inherent variability. The source of this variability may be attributed to many factors, including genetic background differences, culture passage effects, reprogramming artifacts, and perhaps inherent variability in how efficiently each line responds to differentiation activators. Neuronal heterogeneity seems to amplify this. Cortical neurons are one of the most diverse cell populations in the body. Moreover, recent advances in single-cell technologies suggest that there may be hundreds of distinct neuronal subtypes in the cortex, far more than the traditional handful of categories [36]. This heterogeneity appears to be fundamental to how cortical circuits process information, with different neuron types contributing specialized computational functions to overall network behavior.

The literature on iPSC-based models of ASD repeatedly documents aberrant Ca^2+^ signaling as a potential contributor to the atypical developmental processes in ASD. Many of these studies, however, were directed toward examining spontaneous Ca^2+^ transients and/or studying specific ASD mutations or risk variants. Avazzadeh et al. (2019) [16] investigated spontaneous Ca^2+^ signaling of cortical neurons derived from iPSCs that harbor ASD-relevant mutations in *NRXN1*. They showed that ASD iPSC-derived cortical neurons had higher frequency of Ca^2+^ transients, with longer duration and bigger amplitude. Teles et al. (2022) [17] investigated iPSCs harboring ASD risk variants in the *RELN* and *CACNA1H* genes. They showed that ASD-associated variants in the *CACNA1* and *RELN* genes caused an increased influx of Ca^2+^ into the neural progenitors and an abnormal migration phenotype. Using the organoid system, Paulsen et al. (2022) [20] investigated three haploinsufficiencies in three ASD risk genes, *SUV420H1*, *ARID1B*, and *CHD8*, and reported asynchronous development of the organoids, accelerated neuronal differentiation, and reduced spontaneous Ca^2+^ transients. Most recently, Shin et al. (2023) [18] showed that knockout of ASD risk gene *TRPC6* in iPSC-derived neurons caused dysregulated Ca^2+^ homeostasis, leading to hyperexcitability of neurons.

Calcium signaling follows a precise developmental timeline that is critical for normal neural development, where specific calcium kinetics are required for normal development [37,38,39]. Disruptions at any stage cause lasting consequences. Components like parvalbumin, which is a slow calcium buffer [40], undergo developmental changes that affect calcium kinetics. In addition, *Ip3r2* knockout mice exhibit ASD-like behaviors, creating developmentally inappropriate calcium kinetics. Our transcriptomic data and others [14,15] revealed differential expression of calcium-handling genes in ASD patients. DeRosa et al. (2018) [14] examined transcriptional differences between iPSC-derived cortical neurons of idiopathic ASD and controls at two time points along the course of differentiation, including day 35 and day 135. Their findings revealed that calcium signaling pathways were dysregulated, and this dysregulation was particularly evident in day 35 of the developmental time course. They also provided functional evidence whereby ASD neurons displayed reduced spontaneous Ca^2+^ transients and less activity on microelectrode array recordings compared controls [14]. Li et al. (2021) [15] conducted transcriptomic analyses using non-syndromic ASD iPSCs and performed their studies in samples from different stages along the differentiation process. Their analysis revealed that many ASD target genes are enriched in the Ca^2+^ signaling pathway, and this was detected across both neural progenitor and mature neurons. Altogether, these studies support the notion that disrupted Ca^2+^ homeostasis emerges early during neurogenesis and may act as a contributing factor in the abnormal developmental processes in ASD.

Our pharmacological characterization of receptor-mediated Ca^2+^ release showed that differentiated cortical neurons had a reduced maximum Ca^2+^ response to ATP in comparison to iPSCs and displayed strong responses to KCl and DHPG. We used ATP at the iPSC stage during initial scouting to ascertain whether the difference in calcium DEG transcriptomics was associated with a fundamental difference in real-time calcium signaling. Calcium transients are clear examples of biological computation, involving triggers, dynamic amplification, intracellular store release, extracellular influx, store recharging, intracellular buffering, and mitochondrial buffering, which in turn integrate calcium signals with the cell’s metabolic states. This interconnected network involves more players because cells express different receptors as they differentiate and mature. Neurons are excitable cells, hence, during their differentiation and maturation, they upregulate voltage-gated channels, ligand-gated channels, and all associated machinery required for their specialization. It is therefore likely that the different Ca^2+^ responses to specific stimuli in Diff neurons compared to iPSC Cells reflect their cellular specialization. It is well established that KCl depolarizes neurons and induces Ca^2+^ influx via voltage-gated Ca^2+^ channels [41], while DHPG activates mGLURs and induces mobilization of Ca^2+^ from internal stores via the phospholipase C beta/IP3 pathway [42]. These signaling pathways are vital for neuronal excitability, synaptic neurotransmission, and modulation of synaptic functions.

We observed reduced Ca^2+^ responses to ATP at the Diff stage compared to the iPSC stage, which may reflect developmental changes during neurogenesis. ATP activates purinergic receptors, including ionotropic P2X and metabotropic P2Y subtypes, leading to Ca^2+^ influx either directly through channel receptors or indirectly via internal stores through the phospholipase C beta/IP_3_ pathway, depending on the receptor subtype. Purinergic signaling has been shown to play a critical role in regulating the proliferation, survival, and differentiation of various cell types, including stem cells and neural progenitors [43,44,45]. During early neurodevelopment, ATP and purinergic signaling are known to influence the migration of neural precursors and intermediate progenitor cells [44,46]. However, as these cells mature into postmitotic neurons, their responsiveness to ATP diminishes [44], which may explain the attenuated Ca^2+^ responses observed in our differentiated neurons.

Our findings indicated that the differential Ca^2+^ kinetic profiles in response to ATP, KCl, and DHPG across iPSC and Diff neurons were more notable in ASD samples, whereby the ATP-induced maximum Ca^2+^ level at the Diff stage was significantly lower and the responses to KCl and DHPG were significantly higher compared to controls. Adhya et al. (2021) [47] investigated early-stage neurogenesis in heterogeneous ASD iPSCs, revealing atypical differentiation patterns, including abnormal rosette formation, premature maturation, and imbalances in cell fate specification toward excitatory and inhibitory neuronal populations. Paulsen et al. (2022) [20] also showed an accelerated differentiation phenotype specifically in deep layer cortical neurons and asynchronous formation of excitatory and inhibitory neurons in their model organoids harboring mutations in ASD risk genes. Using a whole-cell patch clamp, Hussein et al. (2023) [19] reported early maturation and hyperexcitability of neurons derived from iPSCs harboring mutations in ASD risk genes. In addition, several reports of hyperexcitable phenotypes have been documented in many other studies investigating ASD-specific risk genes and or chromosomal changes [16,18,19]. Future studies should include electrophysiological characterization (patch-clamp/MEA), comprehensive gene expression analysis, and detailed morphometric assessments, including synaptogenesis metrics, all of which would provide important functional validation of our findings.

In conclusion, our findings support the notion that calcium dysregulation emerges early during neurogenesis and may act as a contributing factor to abnormal developmental processes in ASD. Future studies may include a larger number of idiopathic ASD iPSC lines to replicate these findings and identify specific molecular targets within calcium signaling pathways that may confer therapeutic potential to restore optimal calcium levels and neuronal excitability.

## Figures and Tables

**Table 1:** Details of iPSC lines used in this study.

Donor ID	Code	Description
007	Control1	Control, 36, Male
FAM	Control2	Control, 58, Male
SC119	ASD1	Idiopathic Autism, 38, Male, no seizures
SC125	ASD2	Idiopathic Autism, 17, Male, no seizures

## Data Availability

The original contributions presented in this study are included in the article/Supplementary Material. Further inquiries can be directed to the corresponding author(s).

## Supplementary Materials

The following supporting information can be downloaded at: https://www.mdpi.com/article/10.3390/cells14171402/s1, Figure S1: Representative immunofluorescence images of pluripotency markers in the SC119 (ASD1) and SC125 (ASD2) iPSC lines. Scale bars = 50 µm; Figure S2: Trilineage differentiation of ASD iPSC lines; Figure S2: (A) Representative immunofluorescence images of mesoderm (α-cardiac actin) and ectoderm (TUBβIII) markers in mesoderm and neuronal progenitors derived from SC119 and SC125 iPSC lines. (B) mRNA expression of endoderm marker GATA6 in cardiac progenitors derived from SC119 and SC125 iPSC lines. Scale bars = 50 μm; Figure S3: (A) Phase contrast images of neural progenitor cells derived from 007, FAM, SC119, and SC125 at the end of the neural induction stage (NI). (B) Representative immunofluorescence images for expression of neural progenitor markers SOX2 and PAX6 in FAM and SC125 neural progenitors. Scale bars = 20 µm; Figure S4: Average expression of (A) telencephalic marker PAX6, (B) forebrain marker FOXG1, and neuronal marker TUBB3 at different stages along the course of differentiation obtained from RNA-Seq analyses. Bars represent the average expression results from two cell lines across three independent experiments (n = 3); Figure S5: Average expression of (A) pyramidal neuron marker EMX1 and (B) layer 6 marker TBR1 at different stages along the course of differentiation obtained from RNA-Seq analyses. Bars represent the average expression results from two cell lines across three independent experiments (n = 3); Figure S6: Representative confocal micrographs in neuronal cultures (labeled with Flou-3-AM) employed in Ca2+ imaging to illustrate intracellular calcium maps in differentiated (one week) control and ASD samples. The calcium changes are color coded so that warm colors indicate high calcium and cold colors indicate low calcium. The color look-up table is set up to show saturated intensities in order to observe low-level staining in axons and dendrites. Frame numbers 0, 44, 229, and 500 in control indicate time zero (before stimulation), approximately at 24 s post-stimulation with ATP (100 µM), 29 s after treatment with the calcium ionophore ionomycin (4 µM), and at the end of the experiments, 100 s after the application of calcium chelator EGTA (1 mM), respectively. Frame numbers, 0, 74, 271, and 500 in ASD micrographs indicate time zero (before stimulation), approximately 54 s post-stimulation with ATP (100 µM), 71 s after treatment with the calcium ionophore ionomycin (4 µM), and at the end of the experiments, 100 s after the application of calcium chelator EGTA (1 mM), respectively; Figure S7. RNA samples qualitative and quantitative testing report; Table S1: List of DEGs at the iPSC stage; Table S2: List of DEGs at the Diff stage.
