# Supplementary material for: Autism Spectrum Disorder Induced Pluripotent Stem Cells Display Dysregulated Calcium Signaling During Neural Differentiation
Source: Cells. 2025 Sep 8;14(17):1402. doi: 10.3390/cells14171402 (PMC12428247; doi:10.3390/cells14171402)
Supplement: Supplementary file 1 [file cells-14-01402-s001.zip › cells-3827133-supplementary-Tables.pdf]

Table S1. List of DEGs at the iPSC stage.

Gene	log2 (Ipsc_case / Ipsc_control)	Gene	log2 (Ipsc_case / Ipsc_control)
'ZNF717'	9.725383	'PDE12'	1.272002
'EPOP'	2.196837	'DMTN'	3.703605
'CDH4'	1.217206	'AK4'	1.17548
'TMEM178B'	1.257604	'MECOM'	7.253087
'GPC6'	1.279263	'FAT1'	0.82111
'TSHZ1'	3.917809	'UNC5B'	1.257686
'SPRY2'	1.132926	'FBN2'	1.786
'B3GALT5'	1.97384	'EFEMP1'	0.944088
'TUBB3'	1.498353	'FBP1'	2.761973
'HOXB13'	8.037661	'OAF'	2.114782
'FBLN5'	1.859918	'SP8'	2.988558
'POLR3G'	0.843023	'FGF8'	3.247135
'GAS2L1'	1.399787	'CHSY1'	1.26154
'RAB31'	1.071351	'SLITRK3'	1.794074
'CHD3'	1.423976	'BAHD1'	0.768973
'ADAMTS7'	1.063878	'FOXC1'	7.366162
'GLB1L3'	2.0707	'FOXD1'	3.889798
'CHRNB4'	2.205312	'SPART'	0.873324
'FOXP4'	1.621538	'ATP11A'	1.14005
'TMEM266'	1.593598	'SATB2'	2.769232
'CNTFR'	2.806339	'JADE2'	1.446994
'DMBX1'	4.077301	'FN1'	1.076843
'SYT2'	1.486218	'FNTB'	1.779855
'COL4A2'	0.970798	'SLC39A14'	0.831301
'NKAIN4'	1.44209	'MAPK8IP2'	2.165922
'COL6A1'	1.331871	'SEZ6L'	4.133079
'CPT1A'	2.166044	'PATZ1'	0.976937
'GATA5'	4.17968	'SLC7A11'	1.491263
'SIRPA'	1.21948	'CEMIP2'	1.002368
'MANEAL'	1.219697	'IL17RA'	1.387885
'MB21D2'	1.110845	'NR5A2'	2.658461
'ZNF827'	0.973966	'IPMK'	1.327666
'CYP2B6'	2.636842	'GAD2'	4.008121
'ADAMTS16'	1.640189	'MXRA5'	1.931599
'ADAMTS19'	1.081936	'ADGRA2'	2.392246
'DSC2'	1.015639	'TENM4'	1.981842
'DSP'	0.903719	'OSBPL3'	1.180235
'EFNB2'	1.014206	'ATRNL1'	1.341918
'ELAVL2'	1.36771	'SLITRK5'	1.386069
'ABCA2'	1.14438	'TES'	0.98165
'GAS6'	0.95668	'MAN2A2'	1.003511
'GATA2'	3.943061	'MAT2A'	1.394482
'GBX2'	2.503661	'AFF1'	0.943032
'FOXB1'	2.899311	'MLLT6'	1.792739

 https://doi.org/10.3390/xxxxx

Gene	log2 (Ipsc_case / Ipsc_con-trol)	Gene	log2 (Ipsc_case / Ipsc_con-trol)
'NSG1'	1.273946	'MMP17'	1.345129
'CIDEB'	5.248968	'ZNF506'	1.892132
'SALL3'	1.903526	'MT1F'	2.528464
'VENTX'	2.035825	'NAB1'	0.947963
'ZDHHC22'	0.856537	'NBL1'	1.867498
'HID1'	1.257835	'F8A2'	8.906005
'LAMA1'	0.986709	'F8A3'	8.906005
'ZADH2'	1.890011	'NELL1'	5.088636
'ZNF454'	3.897956	'NEFL'	1.65836
'GRIK3'	2.019832	'NFATC1'	1.771063
'HIPK2'	1.253443	'NFIA'	1.937762
'HAS2'	1.774472	'NODAL'	2.989812
'HAS3'	1.398459	'NTRK3'	1.616604
'HMX1'	6.548384	'RNF165'	1.46617
'HPGD'	2.369102	'PAX5'	5.769095
'ARSI'	1.654725	'IRX4'	4.427645
'LRRTM3'	3.686413	'PAX7'	5.02486
'SLCO4C1'	1.424968	'STMN3'	1.000011
'PRAC2'	8.656275	'PCDH1'	0.802378
'ITGA9'	1.550844	'PCSK5'	1.091101
'ITGAV'	0.926531	'CHST15'	1.398975
'ITGB1'	0.800915	'ZNF107'	0.933926
'ITGB5'	1.069403	'PDGFA'	2.056246
'DRAXIN'	0.981505	'GALNT7'	1.173853
'VWC2'	2.386964	'PEX6'	1.208529
'SLC16A12'	2.27785	'ATP8B1'	1.583476
'SHISA6'	2.659575	'CDK14'	1.5491
'RND3'	1.941127	'PITX2'	5.582421
'NKX1-2'	2.111738	'SHC3'	1.566766
'LAMB1'	0.944676	'SLC37A1'	1.467784
'LBR'	0.943279	'SETD4'	1.043108
'TMEM200B'	1.407438	'RIPK4'	1.655194
'LRP3'	0.947112	'SEPTIN5'	2.36004
'LRP4'	1.175711	'CHPF2'	1.332361
'LTBP1'	1.146406	'RBM47'	1.225151
'SMAD7'	1.739405	'SDK2'	1.46641
'POU3F4'	4.287665	'SCN9A'	1.976942
'KIF1A'	1.090533	'SCNN1B'	3.975762
'FAM20A'	1.571597	'PRDM14'	1.103762
'PGPEP1'	1.092825	'PERP'	1.046486
'RPP25'	0.975064	'IRF2BPL'	1.071701
'ZNF770'	1.089225	'MMP25'	1.364464
'PIWIL2'	2.371469	'EBF2'	6.14534
'PPP2R2C'	1.410725	'TNS3'	1.17683
'PPP3CA'	0.818033	'SIX3'	7.810967
'GALNT10'	0.907433	'SKIL'	0.961783
'FERMT1'	1.349802	'SLC1A3'	1.566861
'SEPTIN11'	1.101672	'ZNF649'	1.214848
'LMO3'	1.500629	'BMP7'	1.655625

Gene	log2 (Ipsc_case / Ipsc_control)	Gene	log2 (Ipsc_case / Ipsc_control)
'SULF2'	1.124069	'SLC22A3'	2.230211
'BARX1'	2.191102	'SOX1'	4.158066
'PCDHGB6'	1.517573	'FOXL2'	2.477251
'PCDHAC2'	1.242077	'TBX1'	10.50732
'PRNP'	0.920774	'TBX5'	4.817571
'SERTAD4'	1.88939	'TCF4'	0.946503
'PROS1'	1.785802	'TFAP2C'	1.715355
'AGPAT3'	1.37991	'THBS2'	1.58559
'SPIRE1'	1.157722	'TLL2'	2.017502
'PMEPA1'	1.654665	'ZNF736'	9.366579
'FAM20C'	1.805258	'ZNF726'	8.664644
'STOX2'	1.957221	'WNT5A'	2.348119
'NHSL1'	1.275292	'ZIC1'	6.346009
'PTCH1'	1.452096	'ZNF85'	1.734443
'HEG1'	1.406585	'SLC30A1'	1.644205
'SORCS2'	4.109231	'SLC30A2'	3.642339
'ISLR2'	3.94838	'KCTD15'	1.610871
'TSHZ3'	1.087925	'ATP13A3'	0.846915
'GRHL3'	2.358155	'ARMT1'	1.027745
'PTPRB'	4.325103	'ZDHHC14'	1.516592
'PTPRM'	1.520742	'MCTP1'	2.532394
'PURA'	2.342379	'MOB3B'	0.896884
'PYGL'	0.988785	'LPCAT1'	0.849329
'RHOU'	1.558576	'PLPPR3'	0.878325
'CCND1'	1.627484	'REEP4'	0.926535
'AFAP1'	0.846979	'TSPAN14'	0.756135
'SLC22A23'	1.10156	'KAZALD1'	3.679179
'SLCO5A1'	1.710828		
'NETO2'	1.870049		
'NETO1'	1.46818		
'PCDH11Y'	1.889812		
'CASP3'	1.264828		
'B3GNT5'	1.815519		
'EMILIN2'	1.82015		
'ANTXR1'	1.061934		
'FYTTD1'	1.260159		
'UTF1'	2.75647		
'PRAC1'	8.154713		
'MAML2'	1.35012		
'FBN3'	0.81031		
'TUBB6'	1.740671		
'TNRC18'	2.136463		
'LINGO1'	1.019711		
'DCHS1'	1.280256		
'JMJD7-PLA2G4B'	8.483849		
'GALR2'	4.895459		
'PROM1'	1.285416		
'IER3'	1.912288		

Gene	log2 (Ipsc_case / Ipsc_control)	Gene	log2 (Ipsc_case / Ipsc_control)
'SYNJ2'	2.248506		
'CACNA1H'	1.064679		
'NAV1'	0.925345		
'SAPCD2'	1.236104		
'MIDN'	0.877481		
'PHLDB2'	1.412574		
'SEMA5A'	1.100521		
'ZNF439'	4.506596		
'INA'	1.317558		
'CABLES1'	0.984247		
'DSEL'	1.79795		
'ZNF585B'	6.564792		
'CRLF1'	3.046859		
'ECEL1'	2.350386		
'IGDCC3'	0.933659		
'HS2ST1'	1.123263		
'SPOCK2'	1.656476		
'LRRC37A'	2.773663		
'FGF19'	2.307489		

Table S2. List of DEGs at the Diff stage.

Gene	log2 (diff_case / diff_control)	Gene	log2 (diff_case / diff_control)
'PRR32'	8.486879433	'OSR2'	4.899671643
'ZNF717'	9.253101408	'LRRC3B'	3.460441597
'CDH4'	1.490596858	'C10orf71'	7.717721438
'MRLN'	7.930735315	'SEZ6'	1.53060911
'CDH12'	2.42823105	'JSRP1'	6.241236735
'NBPF19'	0.953385842	'MYOM3'	6.927928947
'HIPK3'	1.171810915	'LRRC39'	3.1190335
'CDH15'	5.142335139	'COL1A1'	1.960211177
'MYMX'	4.927921164	'COL1A2'	2.381062298
'TSHZ1'	1.574213631	'COL3A1'	2.170979227
'LRRC17'	2.052350883	'COL5A1'	2.467412894
'GATD3B'	1.053976337	'COL6A3'	1.920234675
'LOC102724250'	1.357243926	'XIRP2'	8.830428504
'KLHL41'	8.269022576	'COL8A2'	3.23415055
'TRDN'	6.452889262	'COL12A1'	2.541502547
'DLC1'	2.055056675	'COL15A1'	2.282777144
'IFITM3'	1.363728057	'COL16A1'	2.404392511
'SPON1'	2.036274547	'COL19A1'	3.771220059
'CAP2'	0.887154203	'KLHL40'	8.540487053
'ADCY1'	1.807889959	'AFAP1L1'	6.36352716
'ARPP21'	4.094276082	'ASB5'	8.097427527
'TSPAN9'	1.528711397	'SIRPA'	1.654547996
'FGL2'	4.785091095	'CRYM'	2.417680558
'BLCAP'	1.217919599	'RFLNA'	3.534837191
'APOBEC2'	7.732082991	'BEST3'	3.821932192
'VAX1'	5.687853173	'VCAN'	1.48042151
'EMILIN1'	2.891760751	'CSPG4'	2.436163628

Gene	log2 (diff_case / diff_control)	Gene	log2 (diff_case / diff_control)
'LDB3'	3.688103736	'MGAT5B'	0.933947477
'LZTS1'	1.184441425	'UNC45B'	9.94126114
'CHN2'	1.427191239	'HJV'	9.362608068
'CHRNA1'	5.4454839	'SMYD1'	8.802635598
'MGLL'	2.770257273	'FBXO41'	1.458688676
'ADPRHL1'	2.721988566	'ADRA2C'	3.065554479
'CHRNB1'	1.784322905	'SYNE3'	2.927867329
'CHRND'	7.737982914	'DCN'	2.646133381
'CHRNG'	9.131890058	'KANK4'	4.000487821
'CSMD2'	2.080833127	'XIRP1'	6.620125203
'KLHL29'	1.190501122	'DES'	7.529682444
'C1QTNF3'	2.320667345	'SYNPO2'	4.638132288
'CKM'	7.942759086	'DLG2'	1.555144061
'DLX5'	4.238379845	'VGLL2'	8.240485343
'DUSP4'	1.540539119	'STAC3'	4.457694521
'EDNRA'	2.455177937	'EHBP1L1'	2.892752966
'DHRS7C'	7.025192435	'GABRA2'	3.538126088
'EMX2'	3.574822277	'GABRA3'	1.081731691
'ENG'	3.05656538	'NPNT'	3.484631079
'ENO3'	3.785488781	'GABRA5'	2.979447522
'LGI3'	2.737576977	'NEGR1'	0.911685256
'NRK'	6.655837058	'GAD2'	2.355513441
'ERBB3'	5.304797983	'AMBN'	8.281653549
'EYA4'	2.282895023	'PARM1'	1.614488849
'FAP'	2.336325813	'ADGRA2'	2.327368562
'FBLN1'	1.732845893	'MOXD1'	2.341120851
'GJD4'	6.796283675	'CHD5'	2.620314448
'ALDH1A3'	5.618888508	'NUPR1'	2.669084854
'OAF'	1.241850365	'ITGB1BP2'	4.482418154
'RBM24'	1.831721576	'SRPK3'	7.791634228
'FGF7'	3.500011281	'MSTN'	4.784649939
'FGFR4'	3.490029801	'HS6ST3'	1.764463071
'ITGA11'	2.241399555	'GJA5'	4.802130377
'MRAS'	1.233189608	'NSG1'	1.112366443
'FRMPD1'	4.025209165	'HSPB7'	7.991649944
'VASH1'	0.908324249	'IL17B'	10.62861416
'ABLIM3'	2.469599063	'NMRK2'	3.49181689
'FOXC1'	3.132433945	'GP1BB'	1.828274224
'FOXD1'	2.669109174	'GPC1'	0.892487161
'PDZD2'	2.196764166	'ADGRD1'	3.192345658
'MYT1L'	1.708402793	'GPR12'	2.488978292
'PEG10'	0.917191186	'FBXL22'	3.474829992
'TMEM131L'	0.883647452	'GPR21'	2.805775838
'ICOSLG'	3.379507692	'GPR26'	3.185694288
'NEDD4L'	0.881171756	'CCDC141'	5.513714164
'DOCK9'	2.246184168	'C4orf54'	7.720651387
'TNS2'	1.97671377	'ZNF454'	4.590324244
'ATP1B4'	9.033044739	'GRIA3'	1.556670545
'NPTXR'	0.939271554	'GRIK3'	2.029708225

Gene	log2 (diff_case / diff_control)	Gene	log2 (diff_case / diff_control)
'DAAM2'	1.924638717	'GRIN1'	2.878759481
'TTC9'	1.23790009	'GRIN2B'	1.181134087
'SMPX'	10.36504969	'GRM3'	1.058709787
'FLRT2'	1.937061011	'GRM5'	2.348003029
'GUCY1A1'	1.5732749	'MUSK'	3.509409945
'MYLPF'	10.16243914	'MYBPC1'	5.174664768
'ST8SIA5'	2.476121463	'MYBPC2'	9.379382645
'SLC40A1'	1.342977046	'MYBPH'	11.62274944
'CFH'	2.966030129	'MYC'	1.079513403
'HGF'	3.948904915	'MYH3'	8.589259009
'HK2'	1.4611904	'MYH8'	9.205761202
'HMX1'	5.168987218	'MYL1'	9.949327788
'HOXD4'	4.778687937	'MYL4'	8.254890346
'HSPB2'	5.957393155	'MYOD1'	7.543450656
'TNC'	1.785491653	'MYOG'	9.514249373
'STING1'	3.208226859	'NBL1'	2.717080152
'MYLK4'	3.260945311	'NEB'	5.885137415
'SH3RF3'	2.818266483	'F8A2'	8.622124695
'CAVIN4'	1.914783965	'F8A3'	8.622124695
'IGF2'	5.59699472	'NFIX'	2.782418559
'IGFBP4'	1.308178741	'NID1'	1.669779356
'IGFBP5'	1.537835952	'NOTCH2'	0.735368922
'IL11RA'	1.142498851	'ATP2A1'	4.85775718
'ISLR'	3.510036708	'ATP2B3'	1.558480987
'ITGB6'	9.0945319	'DDR2'	1.341650855
'ITIH2'	2.75517089	'TRIM72'	8.401125656
'PEAR1'	2.8349685	'OGN'	4.767494416
'STUM'	2.607920396	'EGFL7'	2.700736243
'KLHL30'	4.621790547	'PCOLCE'	1.744570046
'KCNK2'	1.435814406	'DUSP13'	4.677150032
'SHISA2'	3.383062807	'KCNK9'	2.556685309
'SLC6A17'	1.776282332	'DACT1'	1.153969559
'MYMK'	7.852528536	'PDGFRA'	3.016808559
'LAMA2'	2.373572209	'NSG2'	1.245729823
'LAMA4'	1.697178403	'ASB4'	6.747940526
'LGALS1'	1.794692354	'MYOZ2'	7.810610545
'LMO7'	2.478115415	'PFKFB3'	1.700069676
'KLHL31'	5.643068909	'PITX2'	4.476657013
'ARRB1'	1.432679971	'PITX3'	4.523775364
'MAPT'	0.878679175	'BCL11A'	2.175177872
'MEF2C'	3.423349842	'PLD1'	2.629975314
'MET'	2.634820416	'PRRX1'	2.125901418
'MGP'	4.105000421	'SEPTIN5'	1.449623341
'TMEM151B'	0.764115728	'FBLIM1'	1.633319885
'ZNF562'	2.947171707	'SH3BP2'	0.951794198
'ASPN'	4.088280074	'DEPTOR'	2.338461314
'RPP25'	1.508369333	'ST3GAL1'	1.36953211
'SOHLH2'	3.858256499	'BCL11B'	3.760448366
'PPP1R3C'	2.329712006	'SIX1'	5.792611185

Gene	log2 (diff_case / diff_control)	Gene	log2 (diff_case / diff_control)
'PPP2R2C'	1.854615264	'SLA'	5.364414118
'PRKAR1B'	1.914839809	'REEP1'	1.268521934
'BARX1'	4.161757279	'SLC1A2'	0.822459265
'LMOD3'	10.83647458	'SLC4A3'	1.340986543
'SERTAD4'	1.866914302	'BMP4'	3.526019798
'RELN'	1.160398699	'SLN'	4.757860927
'JPH1'	2.852203794	'RASL11B'	1.531436839
'SEMA3G'	4.61297187	'SNCB'	2.054406349
'FAM20C'	1.218919651	'SPTB'	4.49866375
'JPH2'	4.797895459	'STC1'	2.214271025
'TRIM54'	5.432729685	'SVIL'	2.024776462
'CEMIP'	2.308111518	'TBX1'	10.54914569
'GJD2'	3.422878241	'TBX15'	4.584506369
'RHOJ'	2.564894899	'TCEA3'	3.326048311
'NYNRIN'	1.219420281	'TCF4'	0.949913733
'ACTA1'	9.511862374	'ACTC1'	9.286611445
'MYOZ1'	4.473222105	'TFAP2C'	3.972689633
'ACTA2'	1.222997384	'TGFB3'	1.90045819
'RAPSN'	10.09879495	'TGFBR2'	2.798896249
'PLEKHA2'	1.436684267	'TLE2'	3.499832216
'ALX4'	4.148081014	'TMOD1'	3.082004081
'CELF5'	1.087290637	'TNNC2'	9.07997814
'RXRG'	2.130793076	'TNNC1'	6.22266142
'RYR1'	2.963659711	'TNNI1'	4.865938431
'RYR3'	1.979162733	'TNNI2'	10.33794477
'BGN'	2.013732003	'TNNT1'	5.044520505
'SRL'	5.267546252	'TNNT2'	11.99753303
'PKNOX2'	1.14472304	'TNNT3'	9.08788591
'PGA4'	4.17427274	'TPM2'	3.963541715
'ZNF106'	1.004545534	'TTN'	6.58861946
'WIPF3'	2.01218631	'GXYLT2'	3.497453878
'SGCA'	7.818232081	'EIF3CL'	3.144811659
'SGCD'	3.339575308	'ZNF736'	8.106500674
'SGCG'	2.227935843	'ZNF726'	8.365347757
'CSMD1'	3.018359584	'VEGFA'	1.013004228
'MPPED2'	3.703243712	'CAV3'	10.25603638
'VSNL1'	3.100275498	'STC2'	1.324089925
'XPNPEP2'	3.35491347	'DCHS1'	0.954960964
'MPPED1'	4.147575676	'MYOM1'	4.629749739
'CACNA1A'	2.910706184	'FBP2'	7.630945831
'CACNA1S'	6.293179916	'ACTN2'	5.703971299
'CACNG1'	3.175164018	'INPP4B'	2.434523889
'SEMA3B'	1.695521369	'ALDH1A2'	3.314561014
'TMEM38A'	2.058367085	'HSPB3'	9.411247027
'KCTD15'	1.042618164	'EMILIN3'	1.95859039
'KREMEN2'	2.806240469	'TBX18'	3.203839948
'SYNPO2L'	6.232271898	'COL23A1'	2.734088589
'CPED1'	3.968156426	'ANKRD44'	1.755098779
'SVEP1'	2.149099413	'NEXN'	1.857960212

Gene	log2 (diff_case / diff_control)	Gene	log2 (diff_case / diff_control)
'CSRP3'	9.179642706	'MYOM2'	2.927357245
'ITIH5'	2.927387178	'ZNF804A'	1.401845607
'COL18A1'	1.899025185	'DCLK1'	0.85179359
'CAMK4'	1.046643404	'STYXL2'	7.06590661
'COL21A1'	2.544350144	'ZNF585B'	4.382424881
'CAMK2B'	1.463849305	'C1orf105'	5.945420552
'CABLES2'	1.040802589	'MSC'	4.642747173
'CAPN6'	4.553768231	'OLFM2'	0.894793613
'DYSF'	3.828760628	'ITM2A'	2.754139423
'FZD4'	1.608727656	'SH3BP5'	1.104114972
'FZD8'	3.176474064	'MYOT'	9.530238566
'GLT8D2'	1.907047648	'SH3PXD2A'	1.179635898
'CALN1'	3.092725342	'MICAL2'	1.869087235
'SYT16'	1.419339768	'GPRIN2'	1.493307006
'ZNF528'	1.897342134	'PIEZO1'	2.404050822
'CASQ2'	6.646459516	'MTSS1'	0.840343978
'COL25A1'	3.201187154	'TRIL'	1.813810706
'KIRREL3'	1.228322453		
'MYPN'	8.200837673		
'TRIM55'	7.510507958		
'TRIM63'	5.226878122		
'MYO18B'	6.488734472		
'PPFIA4'	1.568300815		
'ITGA10'	3.175016513		
'SHISAL1'	1.024116393		
'KIAA1755'	3.783006461		
