# Supplementary material for: Autism Spectrum Disorder Induced Pluripotent Stem Cells Display Dysregulated Calcium Signaling During Neural Differentiation
Source: Cells. 2025 Sep 8;14(17):1402. doi: 10.3390/cells14171402 (PMC12428247; doi:10.3390/cells14171402)
Supplement: Supplementary file 1 [file cells-14-01402-s001.zip › Figure S7.pdf]

(1) Sample name: 1A

iPSC_Control_1, RIN=8.2

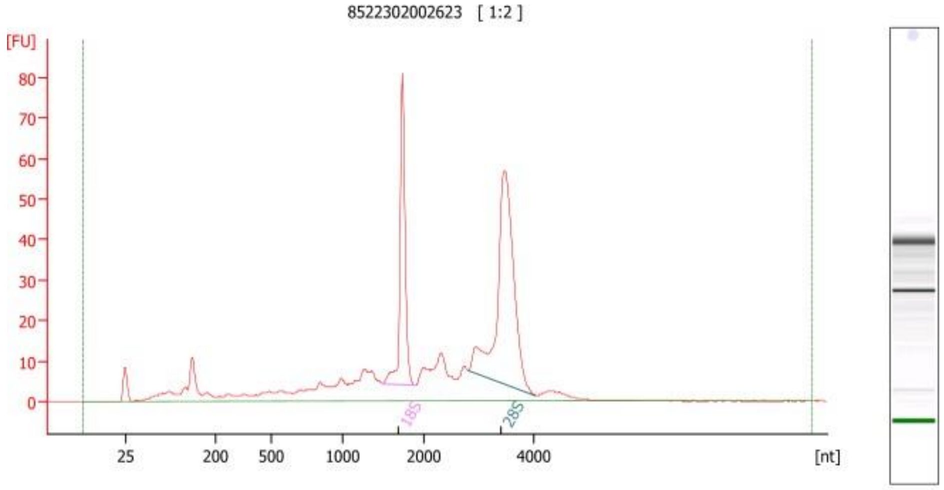

Overall Results for sample 1 : 8522302002623

RNA Area:	499.3	RNA Integrity Number (RIN):	8.2 (B.02.11)
RNA Concentration:	474 ng/µl	Result Flagging Color:	
rRNA Ratio [28s / 18s]:	1.8	Result Flagging Label:	RIN: 8.20

Fragment table for sample 1 : 8522302002623

Name	Start Size [nt]	End Size [nt]	Area	% of total Area
18S	1,483	1,884	76.7	15.4
28S	2,803	4,016	141.5	28.3

(2) Sample name: 2A

NI_Control_1, RIN=9.7

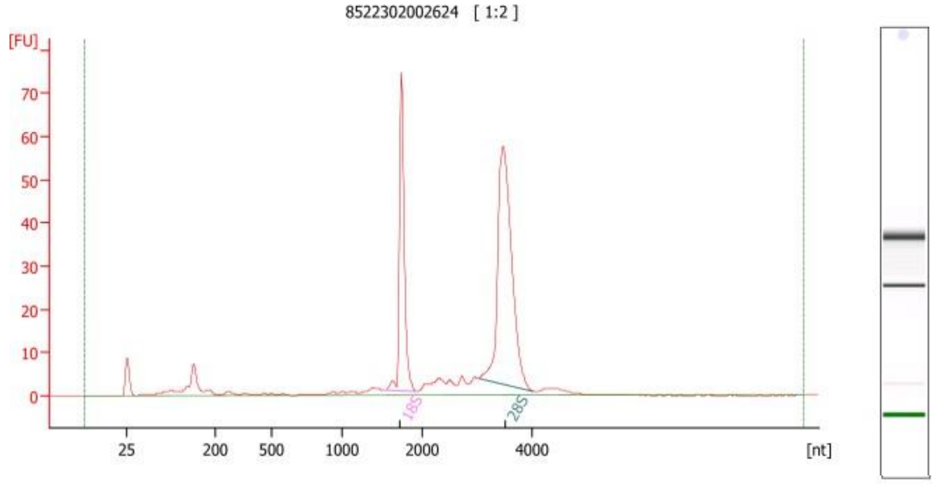

Overall Results for sample 2 : 8522302002624

RNA Area:	301.5	RNA Integrity Number (RIN):	9.7 (B.02.11)
RNA Concentration:	286 ng/µl	Result Flagging Color:	
rRNA Ratio [28s / 18s]:	1.8	Result Flagging Label:	RIN: 9.70

Fragment table for sample 2 : 8522302002624

Name	Start Size [nt]	End Size [nt]	Area	% of total Area
18S	1,530	1,921	70.9	23.5
28S	3,029	4,040	125.4	41.6

(3) Sample name: 3A

NSP_Control_1, RIN=9.6

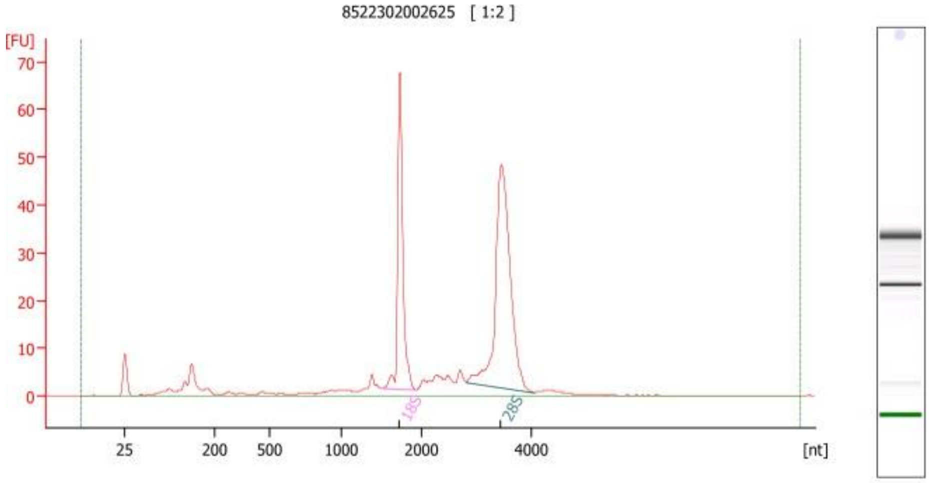

Overall Results for sample 3 : 8522302002625

RNA Area:	290.6	RNA Integrity Number (RIN):	9.6 (B.02.11)
RNA Concentration:	276 ng/µl	Result Flagging Color:	
rRNA Ratio [28s / 18s]:	1.7	Result Flagging Label:	RIN: 9.60

Fragment table for sample 3 : 8522302002625

Name	Start Size [nt]	End Size [nt]	Area	% of total Area
18S	1,507	1,923	67.7	23.3
28S	2,815	4,046	113.1	38.9

(4) Sample name: 4A

Diff_Control_1, RIN=9.4

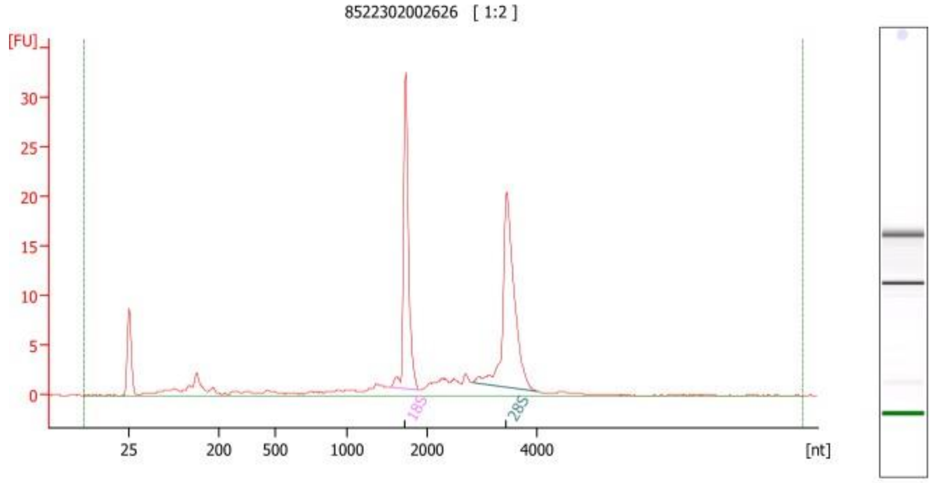

Overall Results for sample 4 : 8522302002626

RNA Area:	119.4	RNA Integrity Number (RIN):	9.4 (B.02.11)
RNA Concentration:	113 ng/µl	Result Flagging Color:	
rRNA Ratio [28s / 18s]:	1.2	Result Flagging Label:	RIN: 9.40

Fragment table for sample 4 : 8522302002626

Name	Start Size [nt]	End Size [nt]	Area	% of total Area
18S	1,538	1,916	31.1	26.0
28S	2,840	4,013	37.6	31.4

(5) Sample name: 1B

iPSC_Control_2, RIN=8.90

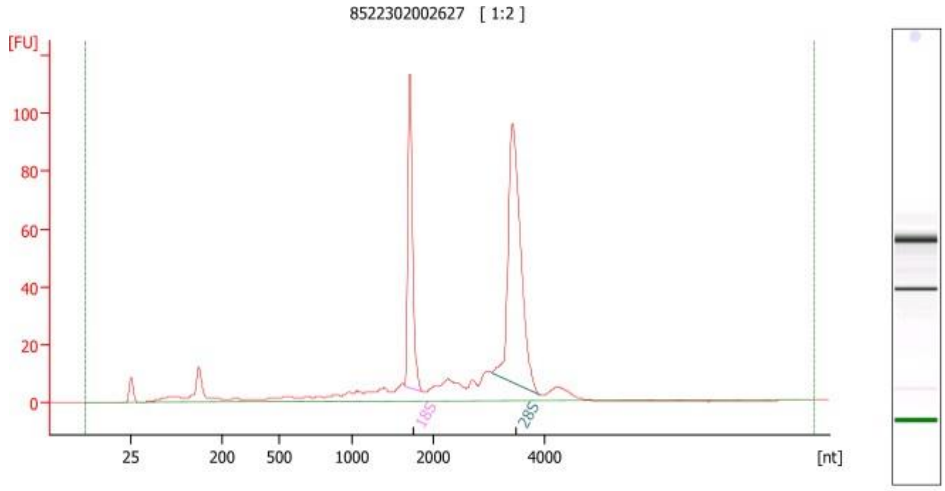

Overall Results for sample 5 : 8522302002627

RNA Area:	527.5	RNA Integrity Number (RIN):	8.9 (B.02.11)
RNA Concentration:	501 ng/µl	Result Flagging Color:	
rRNA Ratio [28s / 18s]:	1.8	Result Flagging Label:	RIN: 8.90

Fragment table for sample 5 : 8522302002627

Name	Start Size [nt]	End Size [nt]	Area	% of total Area
18S	1,646	1,880	99.0	18.8
28S	3,038	3,921	182.6	34.6

(6) Sample name: 2B

NI_Control_2, RIN=9.6

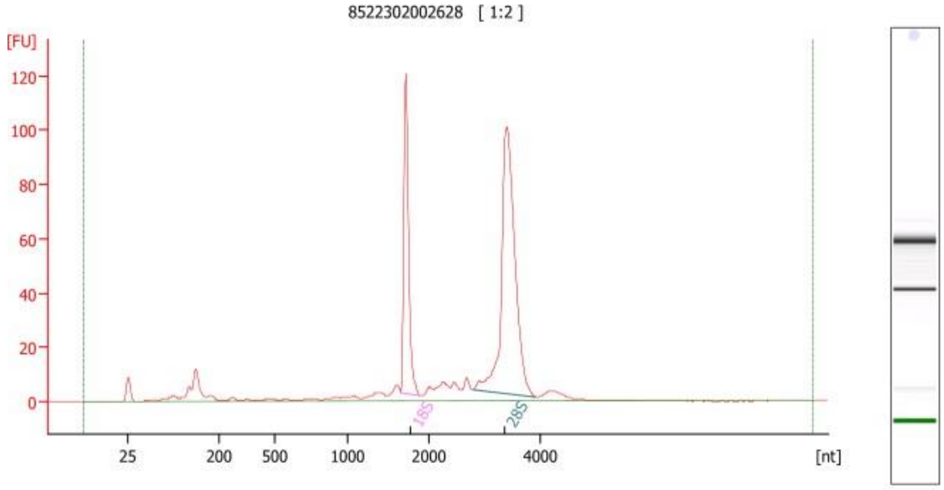

Overall Results for sample 6 : 8522302002628

RNA Area:	499.4	RNA Integrity Number (RIN):	9.6 (B.02.11)
RNA Concentration:	474 ng/µl	Result Flagging Color:	
rRNA Ratio [28s / 18s]:	1.9	Result Flagging Label:	RIN: 9.60

Fragment table for sample 6 : 8522302002628

Name	Start Size [nt]	End Size [nt]	Area	% of total Area
18S	1,651	1,896	110.3	22.1
28S	2,788	3,927	213.9	42.8

(7) Sample name: 3B

NSP_Control_2, RIN=9.6

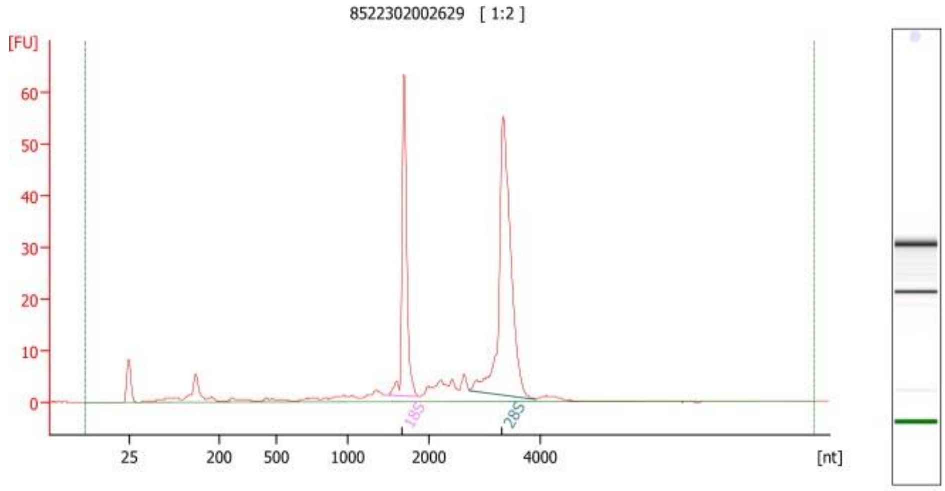

Overall Results for sample 7 : 8522302002629

RNA Area:	245.4	RNA Integrity Number (RIN):	9.6 (B.02.11)
RNA Concentration:	233 ng/µl	Result Flagging Color:	
rRNA Ratio [28s / 18s]:	1.7	Result Flagging Label:	RIN: 9.60

Fragment table for sample 7 : 8522302002629

Name	Start Size [nt]	End Size [nt]	Area	% of total Area
18S	1,468	1,877	59.3	24.2
28S	2,722	3,917	103.5	42.2

(8) Sample name: 4B

Diff_Control_2, RIN=9.3

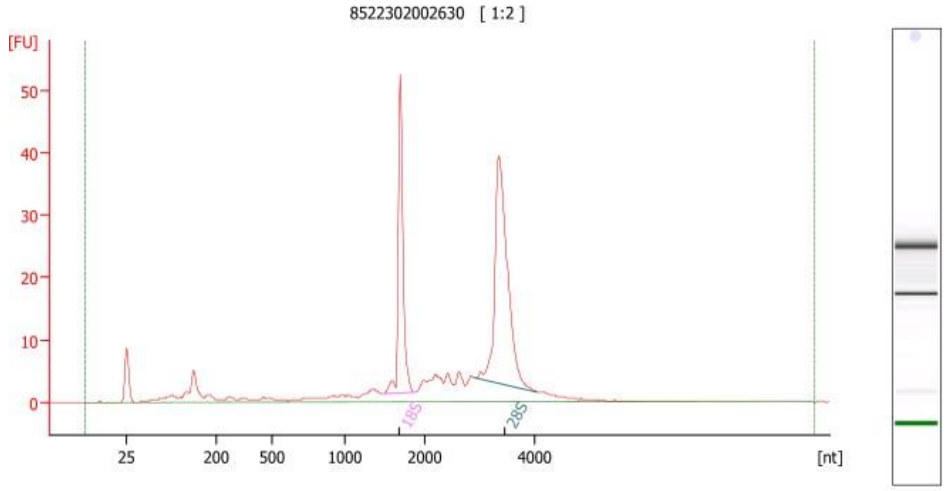

Overall Results for sample 8 : 8522302002630

RNA Area:	241.1	RNA Integrity Number (RIN):	9.3 (B.02.11)
RNA Concentration:	229 ng/µl	Result Flagging Color:	
rRNA Ratio [28s / 18s]:	1.6	Result Flagging Label:	RIN: 9.30

Fragment table for sample 8 : 8522302002630

Name	Start Size [nt]	End Size [nt]	Area	% of total Area
18S	1,481	1,873	48.4	20.1
28S	2,884	4,033	76.8	31.8

(9) Sample name: 1C

iPSC_ASD_1, RIN=9.8

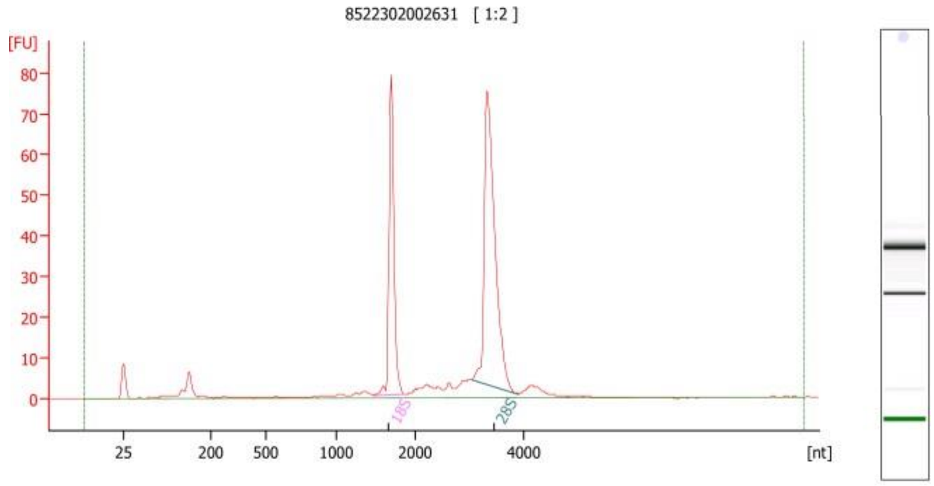

Overall Results for sample 9 : 8522302002631

RNA Area:	298.6	RNA Integrity Number (RIN):	9.8 (B.02.11)
RNA Concentration:	284 ng/µl	Result Flagging Color:	
rRNA Ratio [28s / 18s]:	1.8	Result Flagging Label:	RIN: 9.80

Fragment table for sample 9 : 8522302002631

Name	Start Size [nt]	End Size [nt]	Area	% of total Area
18S	1,465	1,861	74.5	25.0
28S	3,034	3,909	131.2	43.9

(10) Sample name: 2C

NI_ASD_1, RIN=9.4

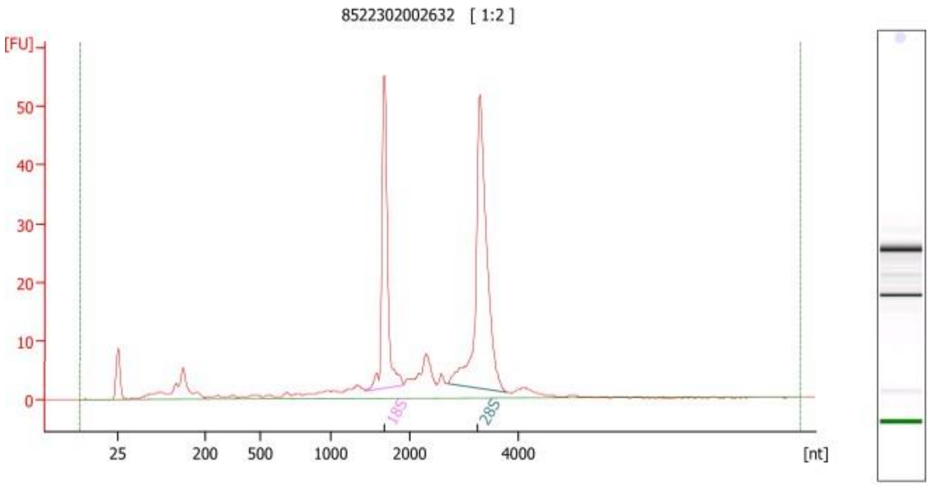

Overall Results for sample 10 : <u>**8522302002632**</u>

RNA Area:	256.3	RNA Integrity Number (RIN):	9.4 (B.02.11)
RNA Concentration:	244 ng/µl	Result Flagging Color:	
rRNA Ratio [28s / 18s]:	1.7	Result Flagging Label:	RIN: 9.40

Fragment table for sample 10 : <u>**8522302002632**</u>

Name	Start Size [nt]	End Size [nt]	Area	% of total Area
18S	1,436	1,924	54.8	21.4
28S	2,698	3,790	95.4	37.2

(11) Sample name: 3C

NSP_ASD_1, RIN=9.1

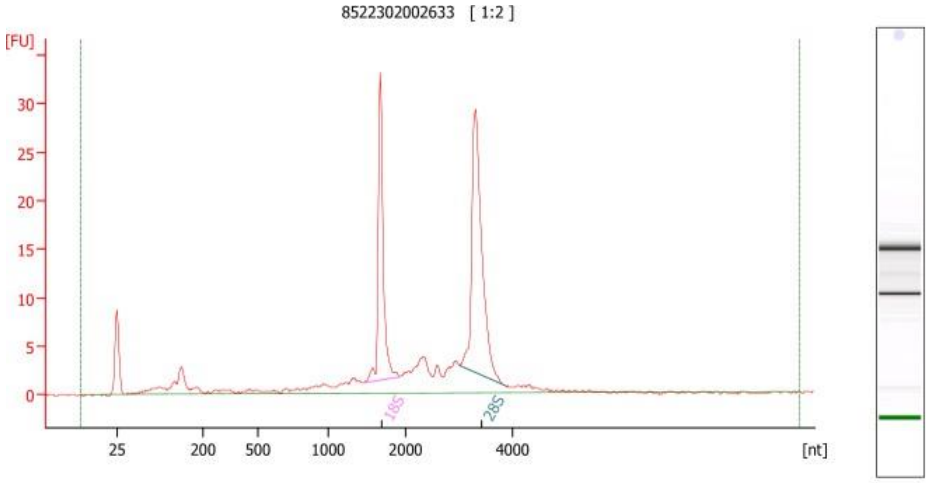

Overall Results for sample 11 : 8522302002633

RNA Area:	155.9	RNA Integrity Number (RIN):	9.1 (B.02.11)
RNA Concentration:	148 ng/µl	Result Flagging Color:	
rRNA Ratio [28s / 18s]:	1.4	Result Flagging Label:	RIN: 9.10

Fragment table for sample 11 : 8522302002633

Name	Start Size [nt]	End Size [nt]	Area	% of total Area
18S	1,448	1,921	31.8	20.4
28S	2,998	3,853	45.5	29.2

(12) Sample name: 4C

Diff_ASD_1, RIN=9.5

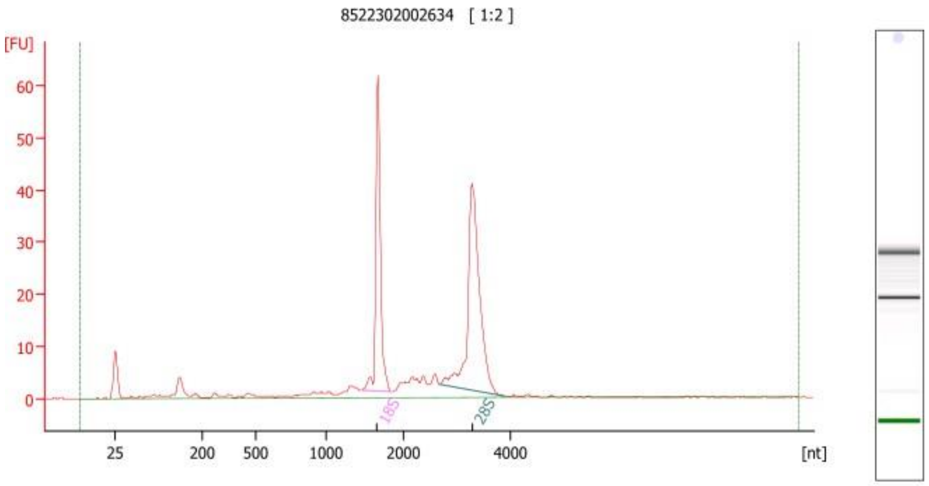

Overall Results for sample 12 : 8522302002634

RNA Area:	218.9	RNA Integrity Number (RIN):	9.5 (B.02.11)
RNA Concentration:	208 ng/µl	Result Flagging Color:	
rRNA Ratio [28s / 18s]:	1.4	Result Flagging Label:	RIN: 9.50

Fragment table for sample 12 : 8522302002634

Name	Start Size [nt]	End Size [nt]	Area	% of total Area
18S	1,455	1,846	57.9	26.5
28S	2,672	3,897	78.8	36.0

(13) Sample name: 1D

iPSC_ASD_2, RIN=9.4

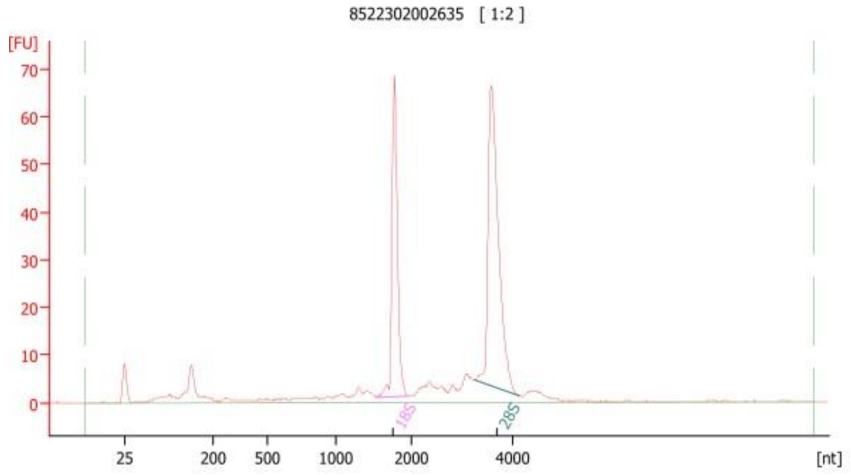

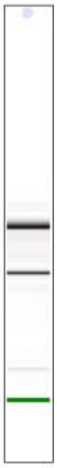

Overall Results for sample 1 : 8522302002635

RNA Area:	310.7	RNA Integrity Number (RIN):	9.4 (B.02.07)
RNA Concentration:	258 ng/µl	Result Flagging Color:	
rRNA Ratio [28s / 18s]:	1.7	Result Flagging Label:	RIN: 9.40

Fragment table for sample 1 : 8522302002635

Name	Start Size [nt]	End Size [nt]	Area	% of total Area
18S	1,527	1,979	66.6	21.4
28S	3,232	4,148	112.3	36.1

(14) Sample name: 2D

NI_ASD_2, RIN=9.4

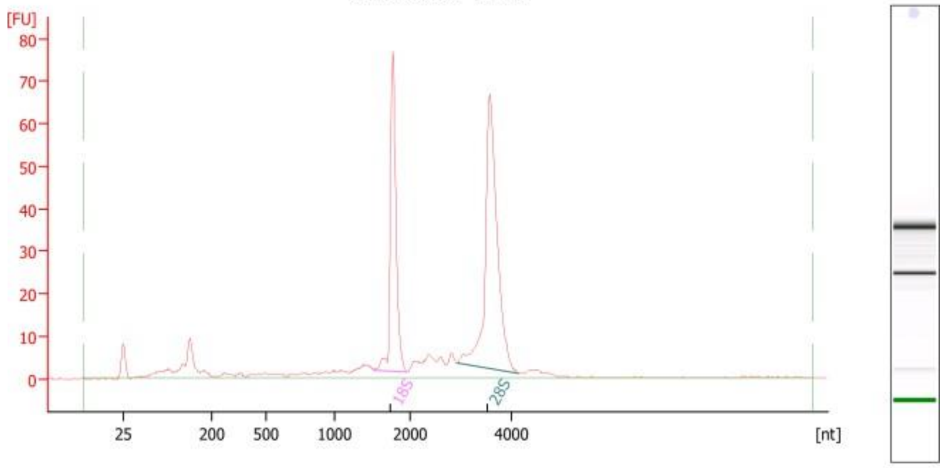

Overall Results for sample 2 : 8522302002636

RNA Area:	355.2	RNA Integrity Number (RIN):	9.4 (B.02.07)
RNA Concentration:	295 ng/µl	Result Flagging Color:	
rRNA Ratio [28s / 18s]:	1.7	Result Flagging Label:	RIN: 9.40

Fragment table for sample 2 : 8522302002636

Name	Start Size [nt]	End Size [nt]	Area	% of total Area
18S	1,517	1,969	77.6	21.8
28S	2,927	4,148	132.4	37.3

(15) Sample name: 3D

NSP_ASD_2, RIN=9.5

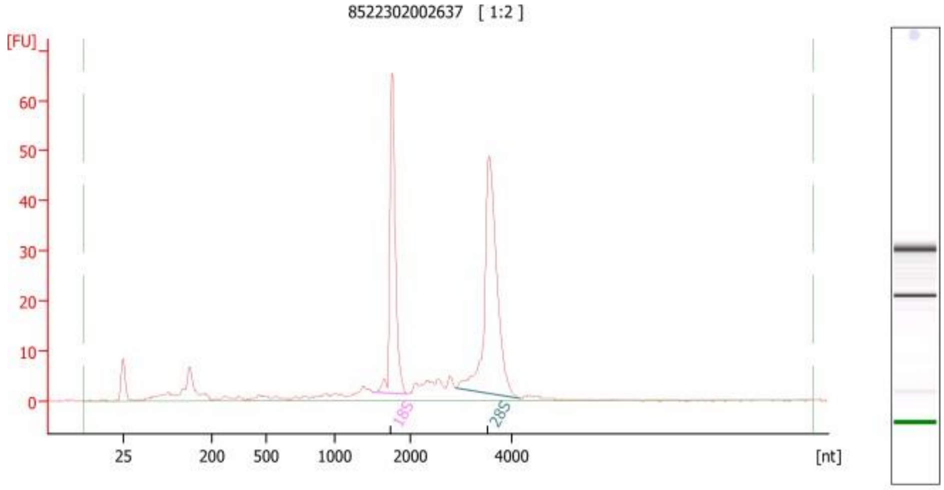

Overall Results for sample 3 : 8522302002637

RNA Area:	275.3	RNA Integrity Number (RIN):	9.5 (B.02.07)
RNA Concentration:	229 ng/µl	Result Flagging Color:	
rRNA Ratio [28s / 18s]:	1.5	Result Flagging Label:	RIN: 9.50

Fragment table for sample 3 : 8522302002637

Name	Start Size [nt]	End Size [nt]	Area	% of total Area
18S	1,517	1,959	65.2	23.7
28S	2,898	4,162	100.9	36.7

(16) Sample name: 4D

Diff_ASD_2, RIN=9.4

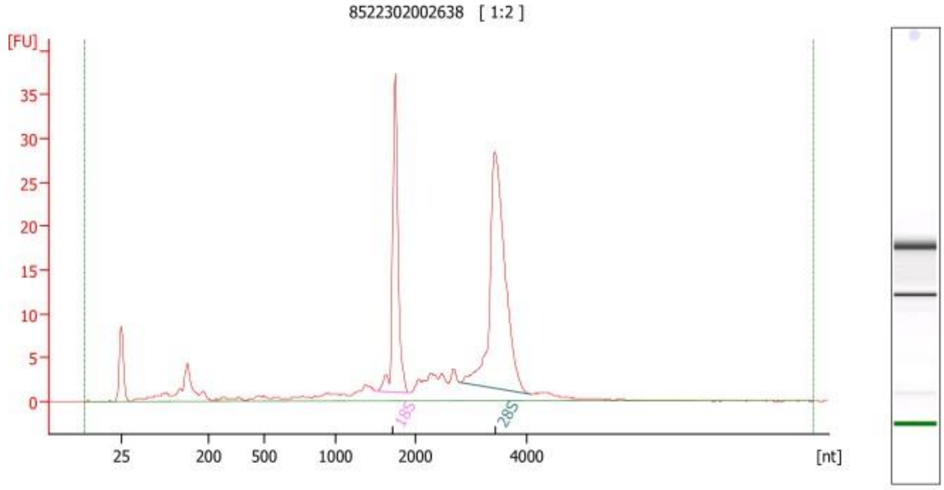

Overall Results for sample 1 : 8522302002638

RNA Area:	191.4	RNA Integrity Number (RIN):	9.4 (B.02.11)
RNA Concentration:	159 ng/µl	Result Flagging Color:	
rRNA Ratio [28s / 18s]:	1.9	Result Flagging Label:	RIN: 9.40

Fragment table for sample 1 : 8522302002638

Name	Start Size [nt]	End Size [nt]	Area	% of total Area
18S	1,510	1,928	37.1	19.4
28S	2,786	4,076	69.6	36.4

Sample Name	Test Instrument	Test Kit	Dilution Ratio(×)	Test Concentration(ng/μL)	Concentration of original sample(ng/μL)	RIN/ RQN	28S / 18S	Remark
1A	Agilent 2100 Bioanalyzer	Agilent RNA 6000 nano Reagents Part 1	3	474	1422	8.2	1.8	
2A	Agilent 2100 Bioanalyzer	Agilent RNA 6000 nano Reagents Part 1	3	286	858	9.7	1.8	
3A	Agilent 2100 Bioanalyzer	Agilent RNA 6000 nano Reagents Part 1	3	276	828	9.6	1.7	
4A	Agilent 2100 Bioanalyzer	Agilent RNA 6000 nano Reagents Part 1	3	113	339	9.4	1.2	
1B	Agilent 2100 Bioanalyzer	Agilent RNA 6000 nano Reagents Part 1	3	501	1503	8.9	1.8	
2B	Agilent 2100 Bioanalyzer	Agilent RNA 6000 nano Reagents Part 1	3	474	1422	9.6	1.9	
3B	Agilent 2100 Bioanalyzer	Agilent RNA 6000 nano Reagents Part 1	3	233	699	9.6	1.7	
4B	Agilent 2100 Bioanalyzer	Agilent RNA 6000 nano Reagents Part 1	3	229	687	9.3	1.6	
1C	Agilent 2100 Bioanalyzer	Agilent RNA 6000 nano Reagents Part 1	3	284	852	9.8	1.8	
2C	Agilent 2100 Bioanalyzer	Agilent RNA 6000 nano Reagents Part 1	3	244	732	9.4	1.7	
3C	Agilent 2100 Bioanalyzer	Agilent RNA 6000 nano Reagents Part 1	3	148	444	9.1	1.4	
4C	Agilent 2100 Bioanalyzer	Agilent RNA 6000 nano Reagents Part 1	3	208	624	9.5	1.4	
1D	Agilent 2100 Bioanalyzer	Agilent RNA 6000 nano Reagents Part 1	3	258	774	9.4	1.7	
2D	Agilent 2100 Bioanalyzer	Agilent RNA 6000 nano Reagents Part 1	3	295	885	9.4	1.7	
3D	Agilent 2100 Bioanalyzer	Agilent RNA 6000 nano Reagents Part 1	3	229	687	9.5	1.5	
4D	Agilent 2100	Agilent RNA 6000 nano Reagents Part 1	3	159	477	9.4	1.9	
