# Supplementary figures and images for: Autism Spectrum Disorder Induced Pluripotent Stem Cells Display Dysregulated Calcium Signaling During Neural Differentiation
Source: Cells. 2025 Sep 8;14(17):1402. doi: 10.3390/cells14171402 (PMC12428247; doi:10.3390/cells14171402)

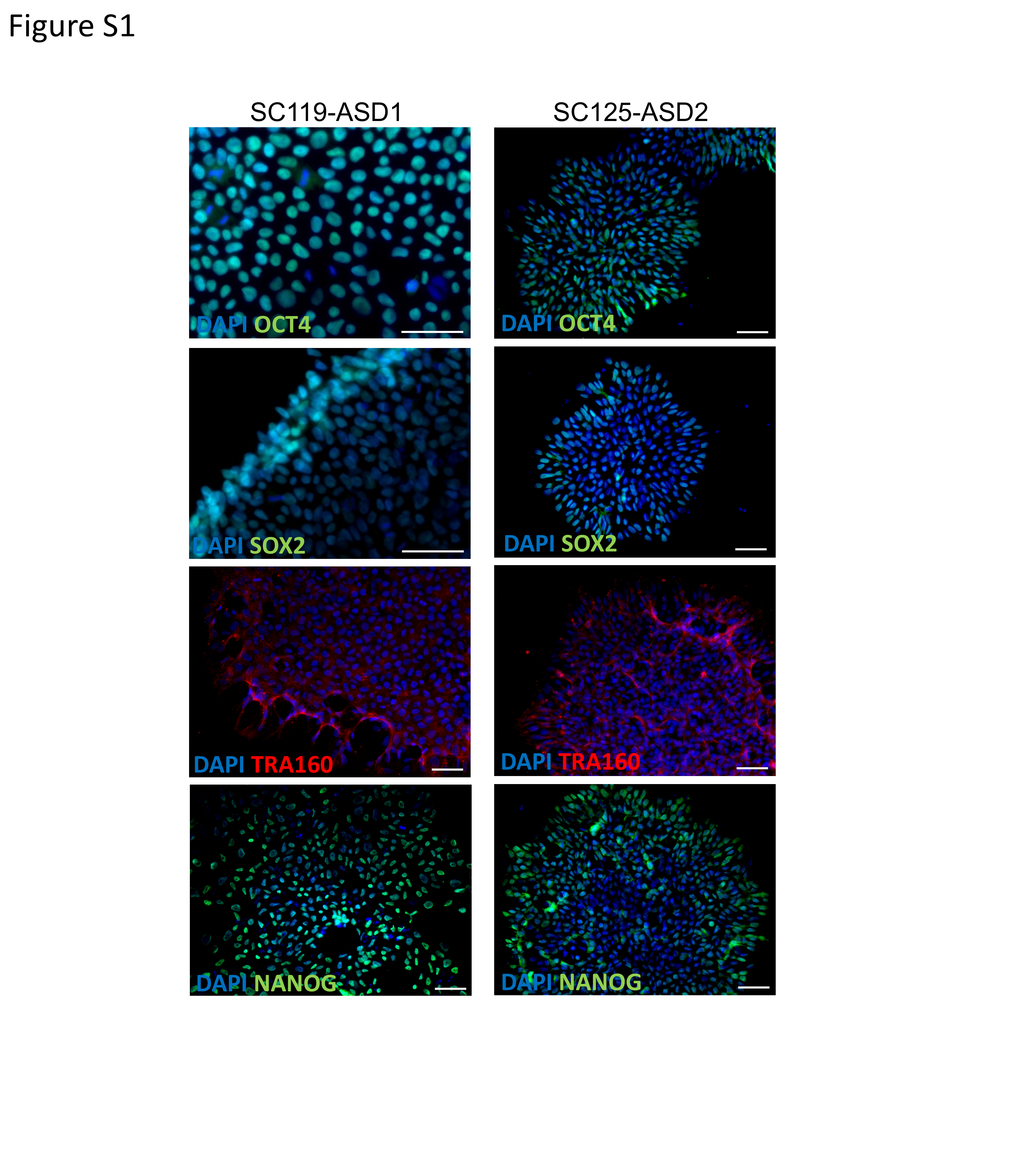

Supplement: Supplementary file 1 [file cells-14-01402-s001.zip › Figure S1.TIF]

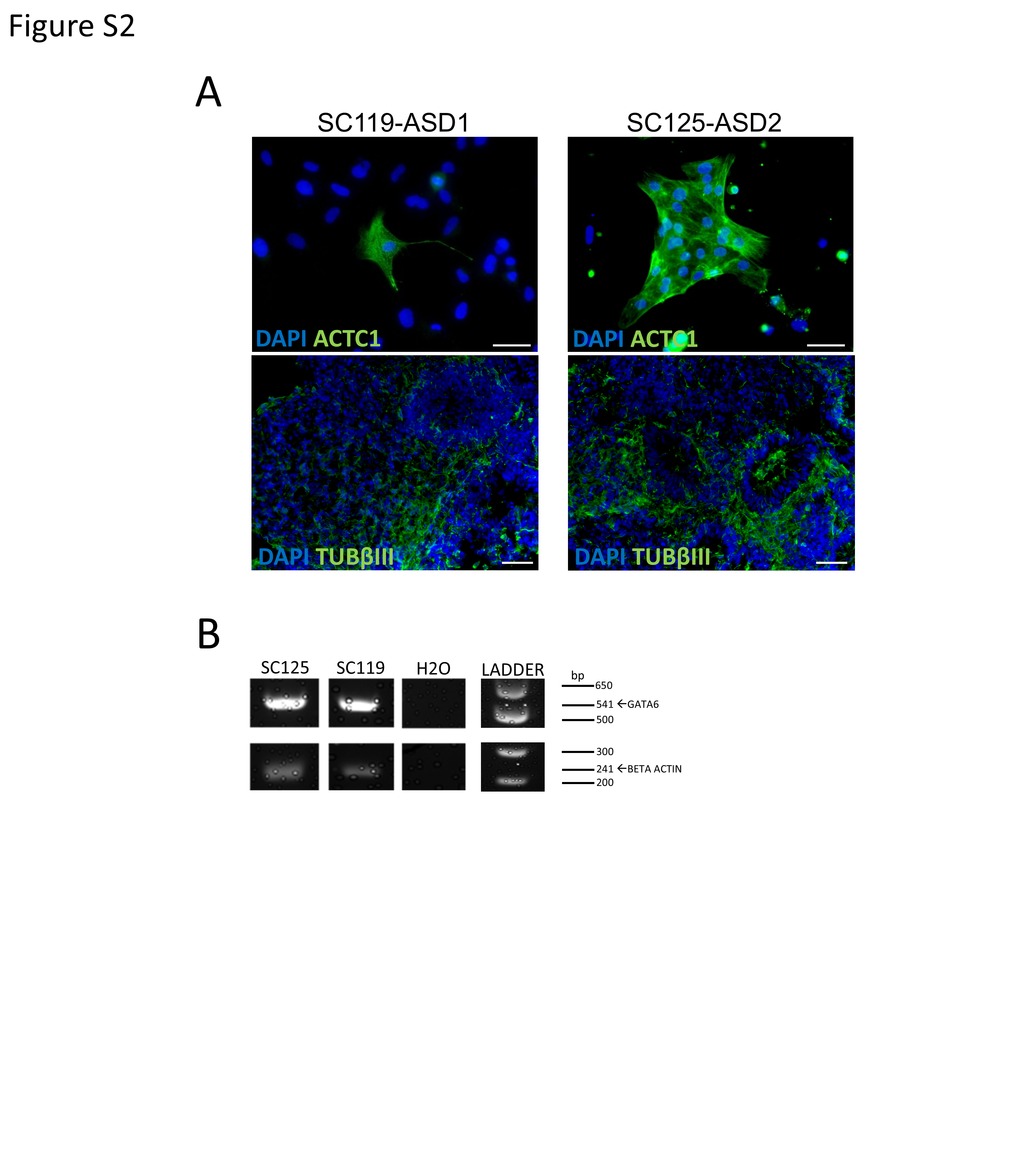

Supplement: Supplementary file 1 [file cells-14-01402-s001.zip › Figure S2.TIF]

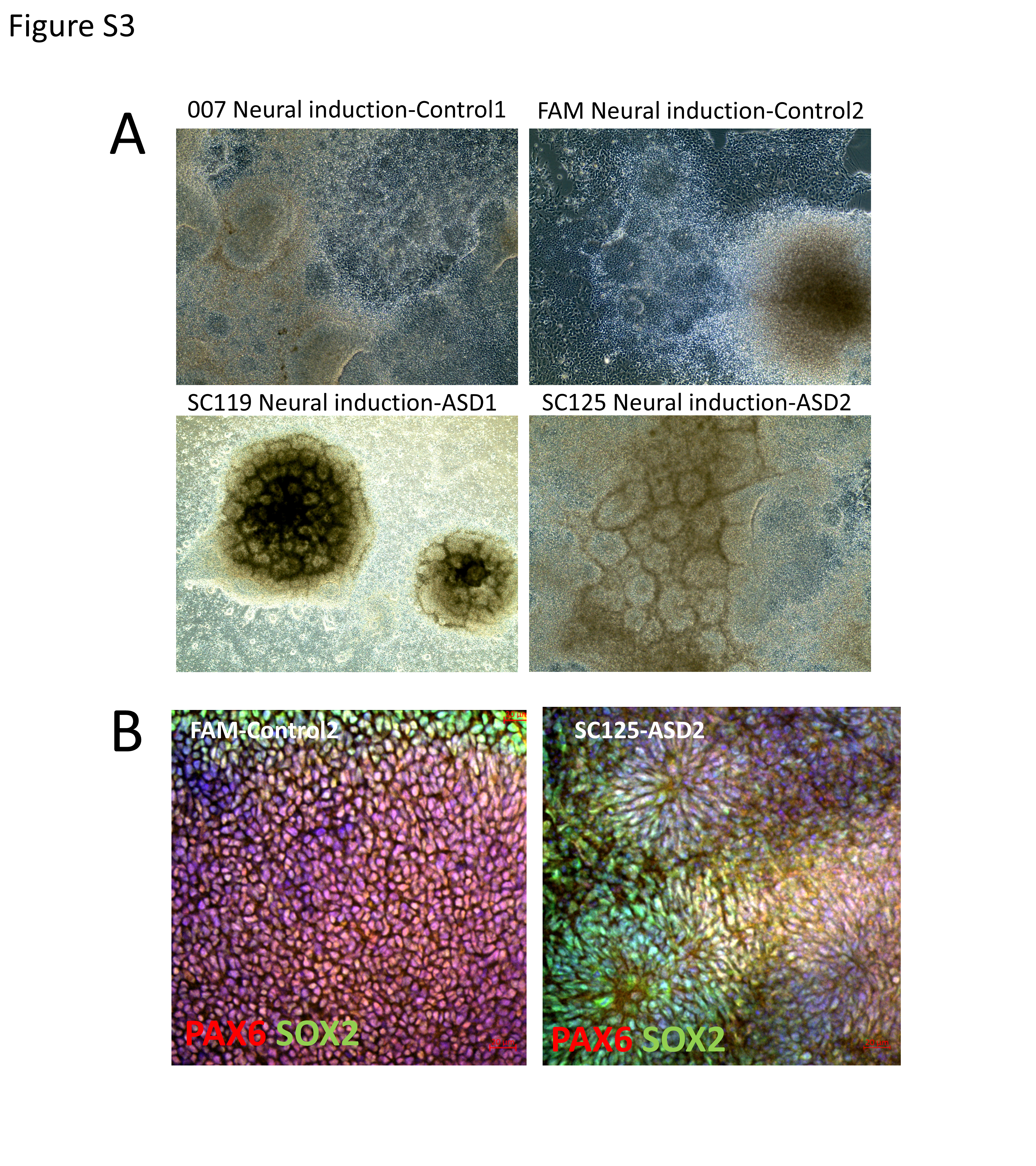

Supplement: Supplementary file 1 [file cells-14-01402-s001.zip › Figure S3.TIF]

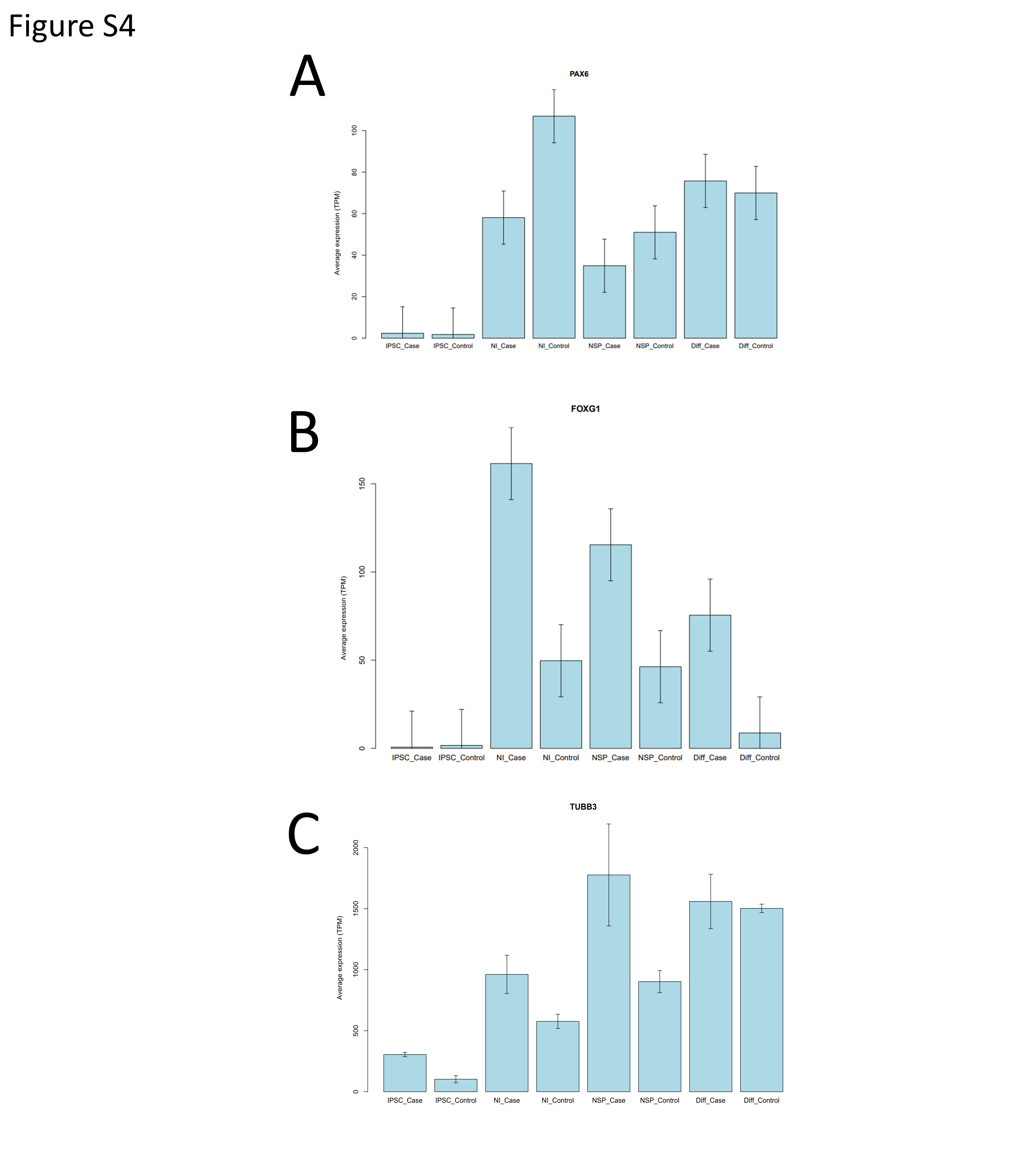

Supplement: Supplementary file 1 [file cells-14-01402-s001.zip › Figure S4.TIF]

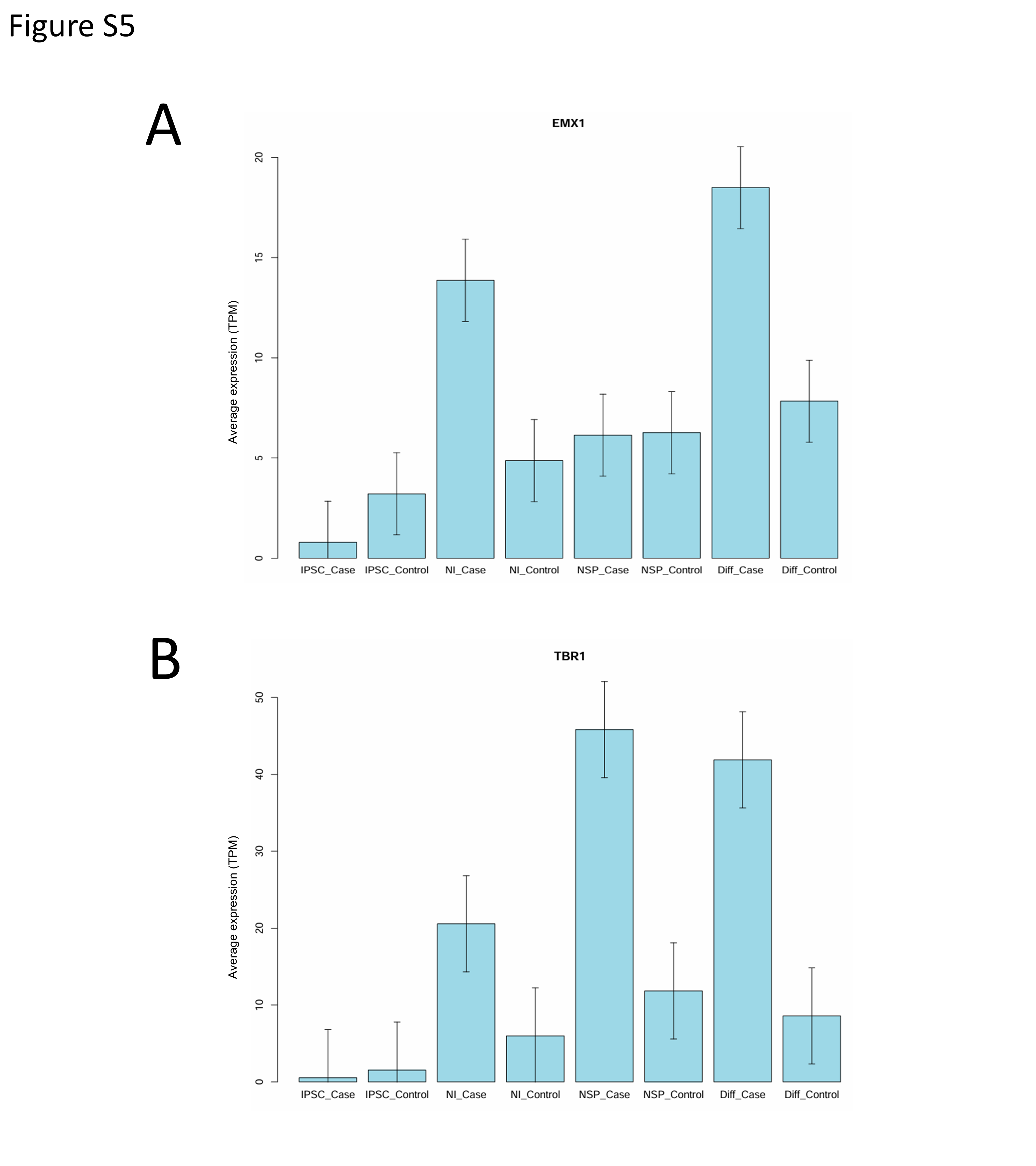

Supplement: Supplementary file 1 [file cells-14-01402-s001.zip › Figure S5.TIF]

Control

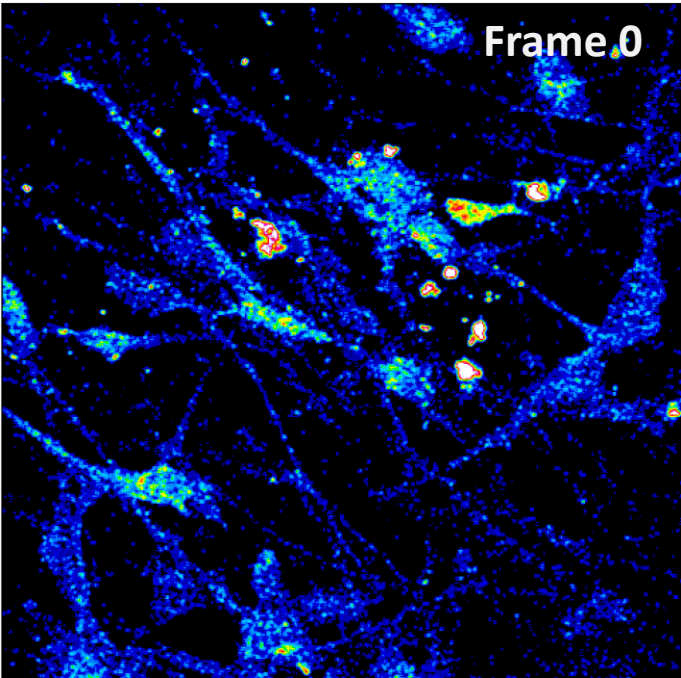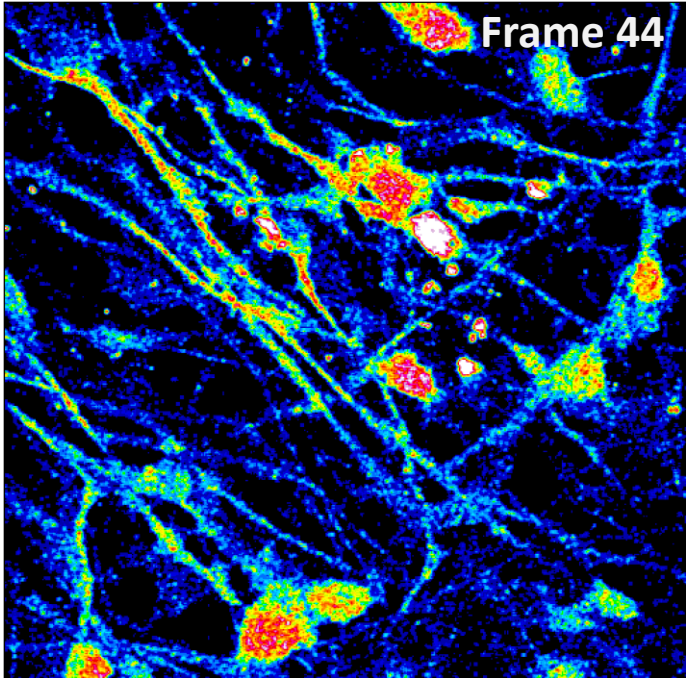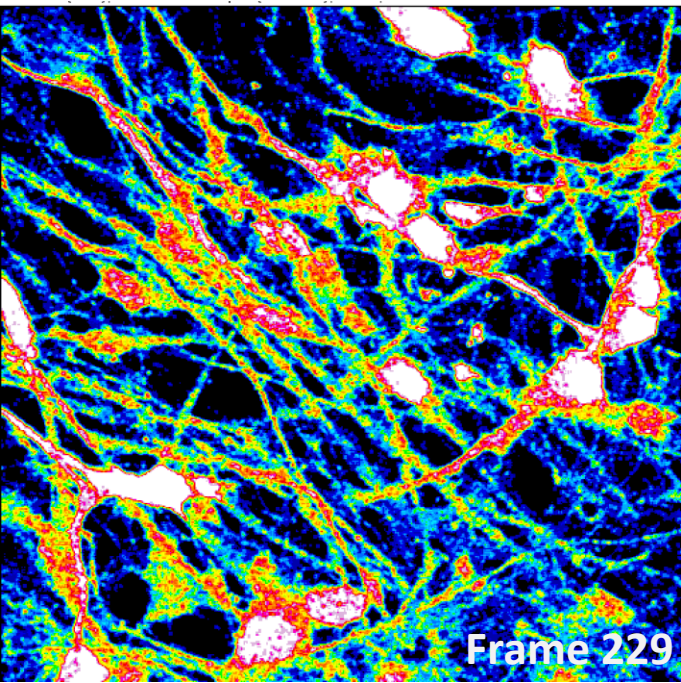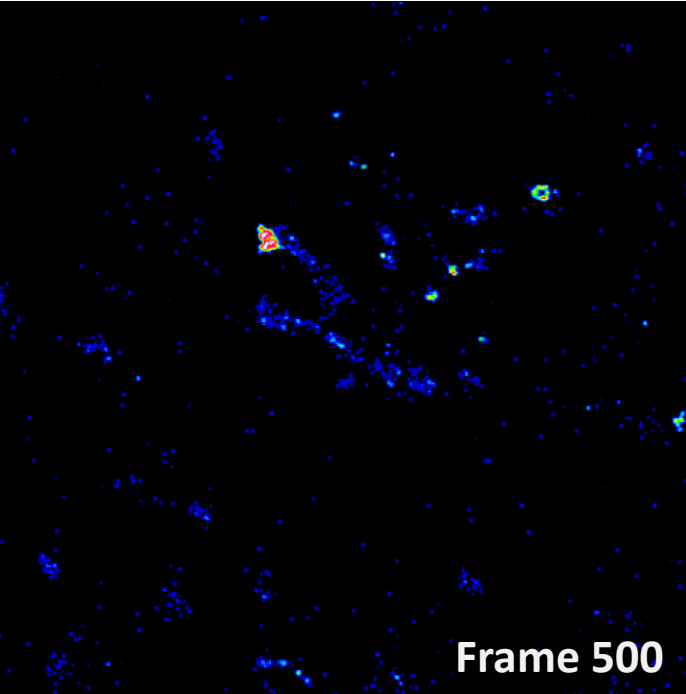

Low

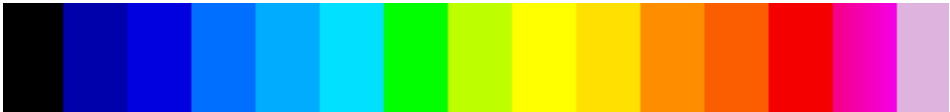

High

ASD

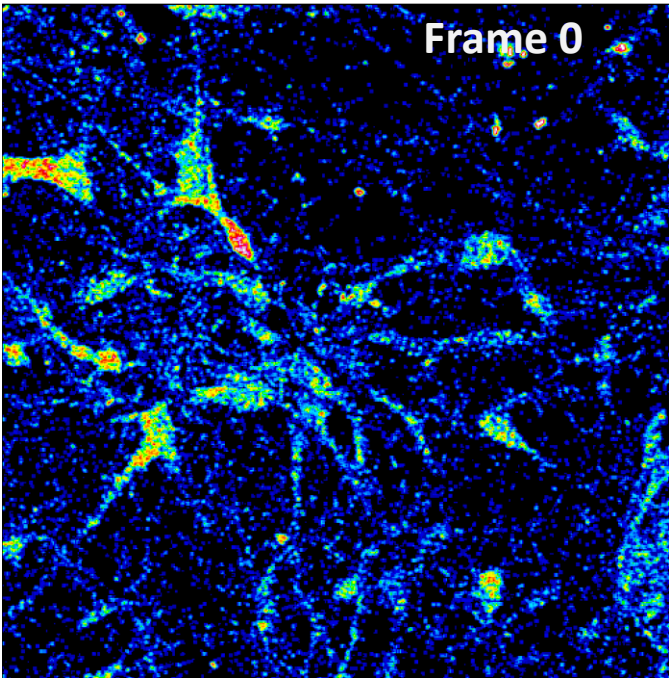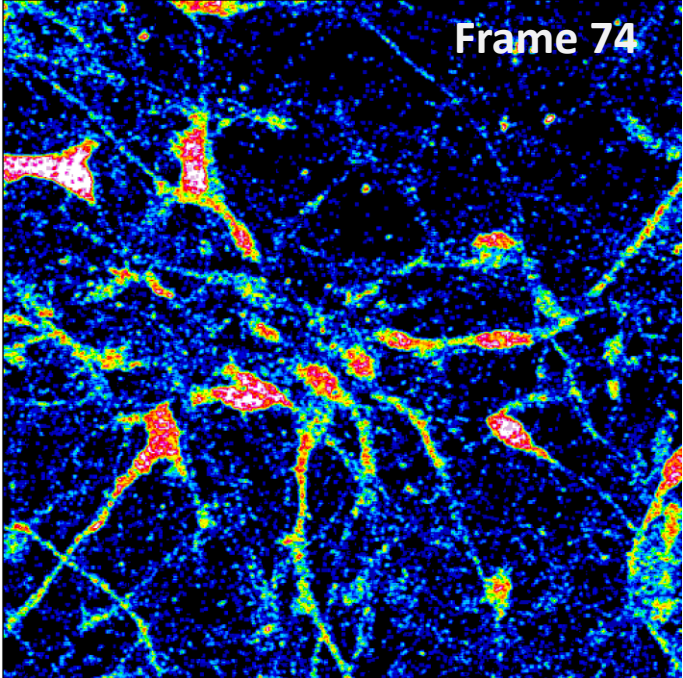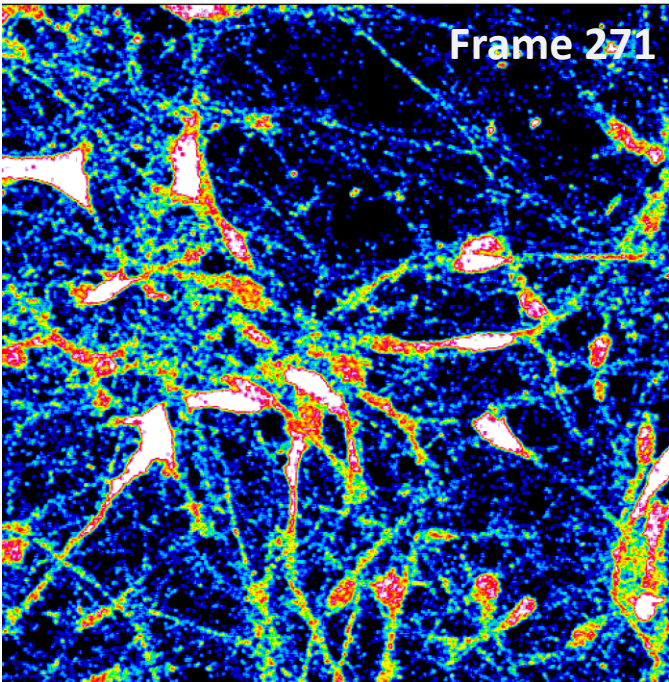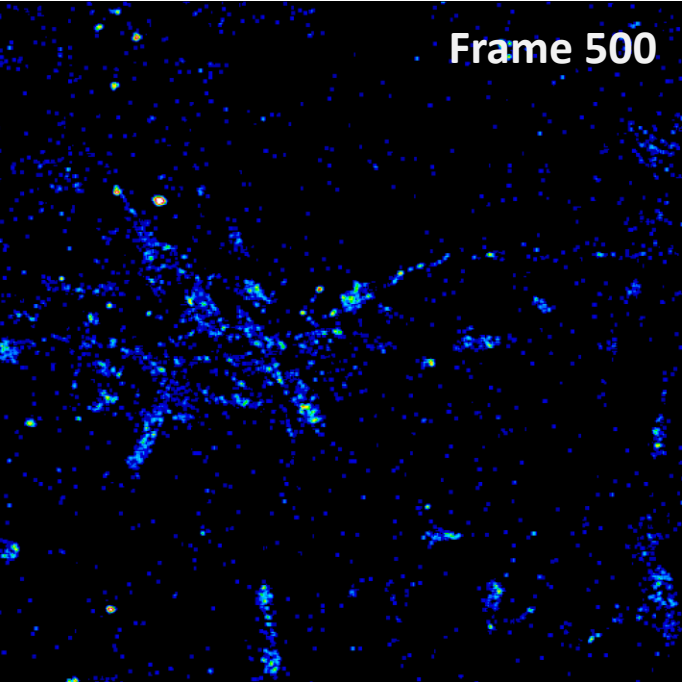

Low

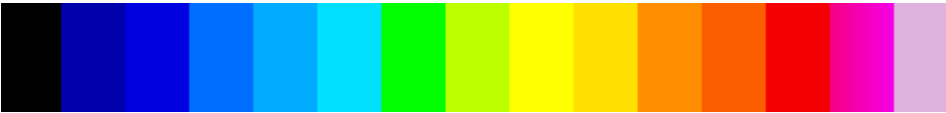

High

Supplement: Supplementary file 1 [file cells-14-01402-s001.zip › Figure S6.pdf]
